# *Armillaria* root rot fungi host single-stranded RNA viruses

**DOI:** 10.1038/s41598-021-86343-7

**Published:** 2021-04-01

**Authors:** Riikka Linnakoski, Suvi Sutela, Martin P. A. Coetzee, Tuan A. Duong, Igor N. Pavlov, Yulia A. Litovka, Jarkko Hantula, Brenda D. Wingfield, Eeva J. Vainio

**Affiliations:** 1 Natural Resources Institute Finland (Luke), Helsinki, Finland; 2 Department of Biochemistry, Genetics and Microbiology, Forestry and Agricultural Biotechnology Institute (FABI), University of Pretoria, Pretoria, South Africa; 3 Laboratory of Reforestation, Mycology and Plant Pathology, V.N. Sukachev Institute of Forest SB RAS, Krasnoyarsk, Russia; 4 Department of Chemical Technology of Wood and Biotechnology, Reshetnev Siberian State University of Science and Technology, Krasnoyarsk, Russia

**Keywords:** Microbiology, Fungi, Pathogens, Virology

## Abstract

Species of *Armillaria* are distributed globally and include some of the most important pathogens of forest and ornamental trees. Some of them form large long-living clones that are considered as one of the largest organisms on earth and are capable of long-range spore-mediated transfer as well as vegetative spread by drought-resistant hyphal cords called rhizomorphs. However, the virus community infecting these species has remained unknown. In this study we used dsRNA screening and high-throughput sequencing to search for possible virus infections in a collection of *Armillaria* isolates representing three different species: *Armillaria mellea* from South Africa*, A. borealis* from Finland and Russia (Siberia) and *A. cepistipes* from Finland. Our analysis revealed the presence of both negative-sense RNA viruses and positive-sense RNA viruses, while no dsRNA viruses were detected. The viruses included putative new members of virus families *Mymonaviridae*, *Botourmiaviridae* and *Virgaviridae* and members of a recently discovered virus group tentatively named “ambiviruses” with ambisense bicistronic genomic organization. We demonstrated that *Armillaria* isolates can be cured of viruses by thermal treatment, which enables the examination of virus effects on host growth and phenotype using isogenic virus-infected and virus-free strains.

## Introduction

The fungal genus *Armillaria* (Fr.) Staude includes more than 40 described species^1^. They are mainly known as notorious plant pathogens of managed natural forests and plantations of non-native tree species that infect hundreds of different plants, including economically important conifers (e.g. *Abies*, *Picea*, *Pinus*) and agronomic plants (e.g. *Citrus*, *Juglans*, *Malus*, *Prunus*, *Vitis*)^2^. Most *Armillaria* species are facultative necrotrophs capable of colonizing living roots, killing root tissues and utilizing dead wood as their source of nutrition. Some species produce edible fruiting bodies, generally known as honey mushrooms. Vegetative diploid individuals (clones) of *A. ostoyae* and *A. gallica* are amongst the largest and oldest organisms on earth, and single clones have been estimated to occupy up to 965 ha and be 1900–8650 years of age^3–6^.

In Europe, the two *Armillaria* lineages causing most damage to trees and woody plants are *A. mellea* and *A. solidipes/ostoyae*^5^, both of which have very broad host ranges^7^. The *A. mellea* lineage has a transcontinental distribution in Europe, North America, Asia and Middle East, and it also occurs as an alien species after being introduced by human activities beyond its natural distribution range. This fungus was introduced into South Africa by the Dutch settlers more than 300 years ago^8^ and has escaped from the planted environment to the sensitive and ecologically important natural woody habitat of the surrounding Table Mountain^9^. In central and northern Europe, *A. ostoyae* occurs sympatrically with *A. borealis* and *A. cepistipes* (syn. *A. lutea*). *A. gallica* is also native to the northern hemisphere (North America, Europe and Japan), where it is considered as a weak pathogen^10–12^. The species also occurs in Kirstenbosch Botanical Gardens in South Africa, presumably being introduced in the country from Asia.

Agricultural crops may be protected against *Armillaria* diseases by root collar excavation or chemical pesticides. Stump removal has been shown as an effective method for controlling *A. ostoyae* in the boreal forest region^13^. However, none of the methods that are presently used can fully eradicate an established *Armillaria* mycelium from a contaminated site.

Fungal viruses (mycoviruses) infect hosts representing diverse fungal taxa and various lifestyles including ascomycetous and basidiomycetous micro- and macrofungi, as well as early-diverging fungal lineages such as Chytridiomycota, Blastocladiomycota, Neocallimastigomycota, Zoopagomycota and Mucoromycota^14–17^. They are also commonly found in edible mushrooms like the cultivated button mushroom (*Agaricus bisporus*) and the shiitake (*Lentinula edodes*) as well as true truffles (*Tuber* spp.)^18–20^. On the other hand, root rot pathogens such as *Heterobasidion* spp. infecting conifers and the white root rot fungus *Rosellinia necatrix* infecting fruit trees have been extensively investigated for the occurrence of mycoviruses^21–23^ in attempts to find viruses suitable for biological control. However, currently the only mycoviral biocontrol agent used in field conditions is the hypovirus CHV1 that significantly reduces the pathogenicity of the ascomycetous Chestnut blight fungus (*Cryphonectria parasitica*) in Europe^24,25^.

Our understanding of the virosphere has recently been revolutionized by high-throughput sequencing (HTS) studies, which have revealed many novel viruses representing yet unassigned virus groups related to but phylogenetically distinct from classified members of the orders *Bunyavirales, Serpentovirales, Hepelivirales, Martellivirales, Tolivirales* and *Tymovirales*, many of which have traditionally known to include only viruses that infect animals or plants (https://talk.ictvonline.org/ictv-reports/ictv_online_report/). Furthermore, metagenomic studies have revealed the common occurrence of newly discovered evolutionary lineages of mycoviruses, such as the viral family *Mymonaviridae* in order *Mononegavirales*^26^, the viral family *Botourmiaviridae* in order *Ourlivirales*^27^, and the unclassified virus group tentatively named “ambiviruses”^28,29^.

Despite their importance as forest and agricultural pathogens, viruses of *Armillaria* spp. have not been molecularly characterized prior to this study. Early studies by Blattný^30^ and Reaves et al.^31^ reported the occurrence of virus-like particles in *A. mellea* and *A. ostoyae*, but these studies were not followed by viral genome sequence characterization. In this study, we used cellulose chromatography and HTS (RNA-Seq) complemented with Sanger sequencing to investigate the occurrence of virus infections in isolates of *A. mellea* and *A. gallica* from South Africa*, A. borealis* from Finland and Siberia and *A. cepistipes* from Finland. In addition, we examined transcriptomic datasets of 12 *Armillaria* species available in GenBank for the presence of viruses using the same bioinformatics pipeline.

## Results

### Virus detection

Initially, we selected 63 *Armillaria* strains for dsRNA virus screening (see Supplementary Table S1 online). The cellulose chromatography analysis did not reveal any clear dsRNA elements in the *Armillaria* isolates, whereas the partitiviruses hosted by the *Heterobasidion* isolates used as control were detected in every assay. Only a very faint fragment of ca. 2 kb was observed in *A. borealis* N40 (data not shown). For this reason, we employed an RNA-Seq approach as an alternative virus detection strategy as it enables the detection all viruses with RNA genomes as well as transcribed DNA viruses. Nine *Armillaria* strains from three different geographical regions (Finland, Russia and South Africa) and three host species (*A. borealis, A. cepistipes* and *A. mellea*) were included in the analysis. The RNA-Seq library of ~ 130 million paired-end reads can be accessed through the BioProject accession number PRJNA685612 in the SRA archive of NCBI.

The de novo contig assembly using Trinity produced a total of 187,685 contigs, of which 64 (see Supplementary Table S2 online) showed sequence similarity with known viruses. Further sequence analysis revealed that they represented seven distinct viruses, some of which were observed in more than one host isolate (Table 1). No amplification was obtained from DNA templates indicating that none of the viruses were integrated into the host genome. Two ORFan contigs (contigs with long ORFs without detectable sequence identity with any known proteins), representing the same sequence, were detected with the criteria used for identification (see Supplementary Table S3 online). None of the viral genomes were 2 kb in size.

**Table 1:** Mycoviruses detected in our collection of *Armillaria* isolates or in transcriptomics datasets deposited in the NCBI SRA database.

Mycovirus	Abbreviation	GenBank ID	BioProject ID	SRA run	Reference	*Armillaria* host strain	Length (nt)	G + C content	Mapping reads^a^	Average depth^b^	Virus with highest Blastx identity^c^	Identity	Query cover	e-value
Armillaria borealis mycovirgavirus 1	AbMV1	MW423800	PRJNA685612	SRR13259820	This study	*A*. *borealis* N40	11,238^d^	43.5%	38,686^d^	344^d^	Auricularia heimuer mycovirgavirus 1^32^	35%	52%	0
Armillaria mellea negative strand RNA virus 1	AmNSRV1	MW423801				*A*. *mellea* CMW3973	10,812	55.1%	35,990	336	Lentinula edodes negative strand RNA virus 1^20^	35%	52%	0
Armillaria mellea ourmia-like virus 1	AmOlV1	MW423802				*A*. *mellea* CMW50256	3,919	53.8%	14,080	348	Agaricus bisporus virus 15^35^	37%	41%	1e-95
Armillaria mellea ourmia-like virus 2	AmOlV2	MW423803				*A*. *mellea* CMW3973	3,162	47.4%	183,835	5718	Apple ourmia-like virus 3 (QIC52830.1)	38%	38%	6e-74
Armillaria borealis ambi-like virus 1^e^	AbAlV1	MW423804				*A*. *borealis* N40	4,975	48.0%	135,187f.	2663f.	Tulasnella ambivirus 4^28^	30%	95%	5e-73
Armillaria borealis ambi-like virus 2^ g^	AbAlV2	MW423806				*A*. *borealis* N40	4,529	49.1%	411,654f.	8880f.	Rhizoctonia solani ambivirus 1^29^	38%	44%	2e-104
Armillaria sp. ambi-like virus 3^ h^	AsAlV3	MW423811				*A*. *borealis* N40	4,521	50.6%	144,486f.	3122f.	Rhizoctonia solani ambivirus 1^29^	36%	44%	1e-104
Armillaria mellea negative strand RNA virus 2	AmNSRV2	TPA: BK014417	PRJNA568830	SRR10392772^i^	JGI, Francis M Martin^j^	*A*. *mellea* ELDO17	11,682	50.2%	484,353^ k^	6855^ k^	Bondarzewia berkeleyi negative-strand RNA virus 1^34^	35%	44%	0
Armillaria ectypa ambi-like virus 1	AeAlV1	TPA: BK014418	PRJNA455898	SRR7968262^i^	JGI, Francis M Martin^j^	*A*. *ectypa* FPL83.16	4,989	47.2%	1,730^ l^	51^ l^	Ceratobasidium ambivirus 1^28^	32%	33%	3e-64
Armillaria luteobubalina ambi-like virus 1	AlAlV1	TPA: BK014419	PRJNA455055	SRR7777675^i^	JGI, Jonathan M Plett^j^	*A*. *luteobubalina* HWK02	4,428	48.8%	5,106	172	Tulasnella ambivirus 4^28^	31%	40%	6e-73
Armillaria mellea ambi-like virus 1	AmAlV1	TPA: BK014420	PRJNA568830	SRR10392772^i^	JGI, Francis M Martin^j^	*A*. *mellea* ELDO17	4,329	47.6%	9,251^ k^	305^ k^	Cryphonectria parasitica ambivirus 1^29^	29%	40%	1e-51
Armillaria mellea ambi-like virus 2	AmAlV2	TPA: BK014421	PRJNA297618	SRR2545913	Tsai et al. ^85^	*A*. *mellea*^m^	4,474	50.6%	14,035	229	Rhizoctonia solani ambivirus 1^29^	37%	44%	1e-104
Armillaria novae-zelandiae ambi-like virus 1	AnzAlV1	TPA: BK014422	PRJNA677795	SRR13091473^i^	JGI, Francis M Martin^j^	*A*. *novae-zelandiae* 2840	4,503	45.7%	1,189	39	Tulasnella ambivirus 4^28^	32%	45%	2e-91

^a^Raw reads were mapped against the virus sequence using Geneious for RNA Seq assembler with medium–low sensitivity.

^b^Mean value generated by Geneious 10.2.6

^c^The sequence having highest identity with the virus based on Blastx with nr database.

^d^Without poly(A) tail.

^e^*A. borealis* Ab9A hosted AbAlV1 variant.

^f^Mapping with custom sensitivity using 0 as a maximum mismatch % per read.

^g^Other *A. borealis* hosts of AbAlV2 were Ab2B, Ab4B, Ab9A and MUS36.

^h^*A. borealis* Ab9A and *A. cepistipes* Al65A hosted AsAlV3 variants.

^i^RNA-seq library was generated using PolyA selection.

^j^Contact person for unpublished datasets of JGI (Joint Genome Institute).

^k^Mapping conducted with custom sensitivity: maximum mismatch 5% per read; maximum gap 5% per read.

^l^Mapping conducted with custom sensitivity: maximum mismatch 1% per read; maximum gap 1% per read.

^m^NCBI GEO: GSM189980.

Publicly available transcriptomic datasets for 13 *Armillaria* isolates representing 12 *Armillaria* species (see Supplementary Table S4 online) were analysed. Three putative viruses were discovered in RNA-Seq libraries of *A*. *mellea*, and one each in *A. ectypa*, *A. luteobubalina* and *A. novae-zelandiae* (Table 1).

### Virus genome characterization and taxonomical assignment

#### Sequence characteristics of a new virga-like virus from a Siberian strain of *A. borealis*

**Figure 1:**
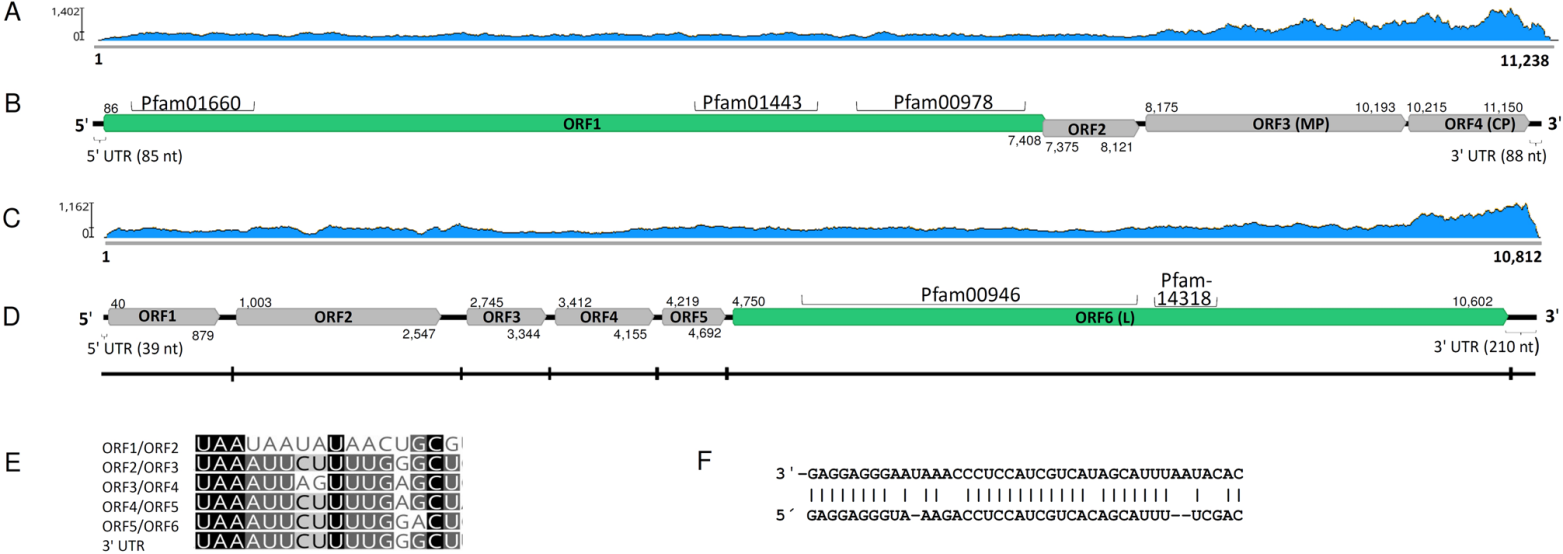
Genome organization of the Armillaria borealis mycovirgavirus 1 (AbMV1) and Armillaria mellea negative strand RNA virus 1 (AmNSRV1). (**A**) Coverage of raw reads mapped against the AbMV1 genome (without poly(A) tail). (**B**) Schematic presentation of AbMV1 genome with UTRs, predicted ORFs and conserved motifs: pfam00978 (RdRP domain, e-value 2.07e−72), pfam01660, (methyltransferase, e-value 1.92e−20) and pfam01443 (helicase, e-value 1.02e−24). Predicted translation initiation and termination sites are marked above or below each ORF. (**C**) Coverage of raw reads mapped against the AmNSRV1 genome. (**D**) Schematic presentation of AmNSRV1 genome with UTRs, predicted ORFs and conserved motifs: pfam00946 (Mononegavirales RdRP, e-value 1.10e−95) and pfam14318 (*Mononegavirales* mRNA-capping region V, e-value 4.82e−16). Predicted translation initiation and termination sites are marked above and below each ORF, respectively. The small vertical lines in the virus genomic segment show the location of the putative gene junction sequences. (**E**) Consensus sequences (uracil-rich tracts) of putative gene junction regions between the predicted ORFs in 3′ > 5′ orientation (genomic RNA). (**F**) Complementarity in the 5′- and 3′-terminal sequences in the genomic RNA strand.

Two nearly identical Trinity contigs shared the highest Blastx similarity with virga-like viruses (see Supplementary Table S5 online). They represented a new virus found to be hosted by *A*. *borealis* strain N40 and designated as Armillaria borealis mycovirgavirus 1 (AbMV1). The 5′ and 3′ untranslated region (UTR) sequences of AbMV1 were determined with Sanger sequencing, resulting in a complete genome sequence of 11,238 nt without the poly(A) tail and four predicted ORFs (Table 1; Fig. 1A,B). Mapping of reads (Table 1) and variant calling using Geneious R10 revealed the sequence to be nearly invariable with only three polymorphic nucleotide sites (see Supplementary Table S6 online). Based on Blastx analysis of the complete genome sequence, the closest relative of AbMV1 was Auricularia heimuer mycovirgavirus 1 (AhMV1, *Martellivirales*; unclassified *Virgaviridae*) from China^32^. The 5′ UTR of AbMV1 includes repeat sequences of UUCCA and UUCAA, which resemble those of the AhMV1. The largest ORF was predicted to encode a replication protein (predicted M_r_ = 272.504 kDa) including an RNA-dependent RNA polymerase (RdRP) domain, a methyltransferase and a helicase (Fig. 1B). The third ORF encodes a protein (predicted M_r_ = 75.310 kDa) homologous to virgavirus movement proteins (MPs), and the fourth ORF resembles a coat protein (predicted M_r_ = 34.078 kDa) similar to those in AhMV1. Interestingly, the second ORF showed some homology with the protease domains of Setosphaeria turcica hypovirus 1 and Phomopsis longicolla hypovirus (GenBank accessions AZT88613 and AIG94930). A recombination event between distantly related viruses (a dsRNA megabirnavirus and a positive-sense ssRNA hypovirus) has been reported earlier by Wang et al.^33^.

**Figure 2:**
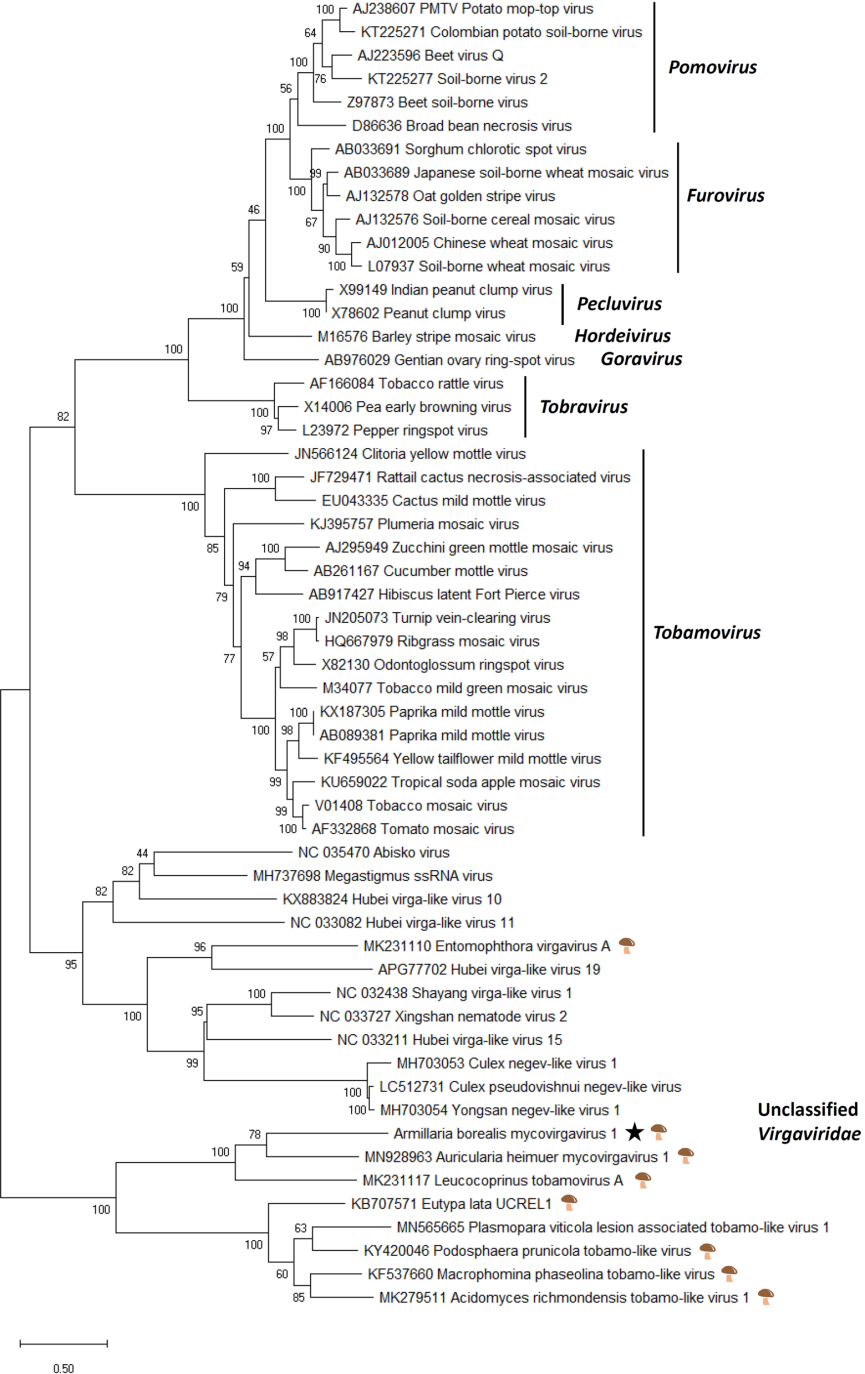
Phylogenetic tree of the family *Virgaviridae* and related unclassified viruses based on an alignment of RdRP aa sequences generated using MAFFT *v7.450* with Blosum45 substitution matrix. The evolutionary history was inferred by using the Maximum Likelihood method and Le_Gascuel_2008 model with 4 gamma categories (+ *G* + *I*). All positions with less than 95% site coverage were eliminated, i.e., fewer than 5% alignment gaps, missing data, and ambiguous bases were allowed at any position. Evolutionary analyses were conducted in MEGA X with 1000 bootstrap repeats^86^. Sequences originating from fungal hosts are indicated with a mushroom symbol and a star denotes the *Armillaria* virus.

Phylogenetic analysis revealed that AbMV1 forms a highly supported cluster with other virga-like viruses from asco- and basidiomycetous fungi (Fig. 2). A separate cluster with unclassified virga-like viruses included Entomophthora virgavirus A (GenBank accession MK231110, unpublished) from a basal fungus (subkingdom Zoopagomyceta) and several viruses obtained from insect metatranscriptomic studies.

#### Putative mymonaviruses hosted by *A*. *mellea* from South Africa and North America

**Figure 3:**
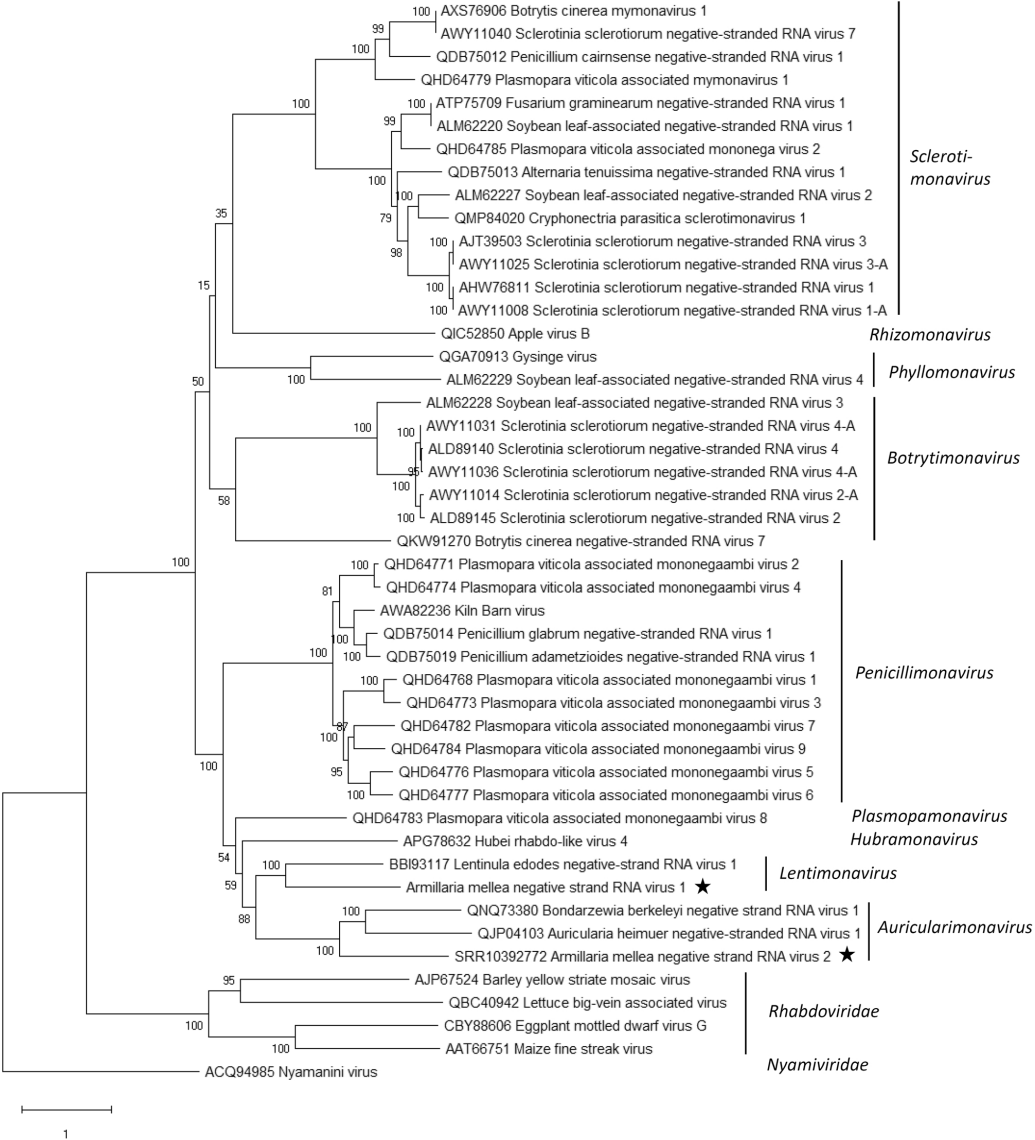
Phylogenetic tree of the family *Mymonaviridae* and selected related families based on an alignment of RdRP aa sequences generated using MAFFT *v7.450* with Blosum45 substitution matrix. The evolutionary history was inferred by using the Maximum Likelihood method implemented in IQ-TREE web server multicore version 1.6.12 at http://iqtree.cibiv.univie.ac.at/^87^. The best-fit model according to ModelFinder was LG + F + I + G4. Bootstrapping was conducted using 1000 repetitions (ultrafast mode). The *Armillaria* viruses are designated with a star.

A new mymona-like virus was identified in the RNA-Seq library of *A. mellea* strain CMW3973 and was represented by two contigs (Table 1; Supplementary Table S5). Mapping of reads (Table 1, Fig. 1C) and variant calling using Geneious R10 revealed that the sequence was nearly invariable with only one polymorphic site (see Supplementary Table S6 online). The terminal 5′ and 3′ UTR sequences were verified by Sanger sequencing, and the virus was named Armillaria mellea negative strand RNA virus 1 (AmNSRV1, Table 1). The complete AmNSRV1 genome is 10,812 nt in length and has six predicted ORFs and 5′ and 3′ terminal UTR regions showing inverted complementarity (Fig. 1C–E). It shared the highest Blastx identity with Lentinula edodes negative strand RNA virus 1 (LeNSRV1)^20^ (Table 1). Furthermore, uracil-rich gene junction sequences were detected in the intergenic non-coding regions of the genomic RNA strand (Fig. 1F) as reported with LeNSRV1^20^. The longest of the predicted proteins in AmNSRV1 contains the Mononegavirales RdRP conserved domain and Mononegavirales mRNA-capping region domain (Fig. 1D). Phylogenetic analysis verified that AmNSRV1 resided in a highly supported cluster together with LeNSRV1, the type species of proposed genus *Lentimonavirus* in family *Mymonaviridae* (Fig. 3; https://talk.ictvonline.org/files/).

Another mymona-like virus was discovered in the SRA dataset from *A. mellea* ELDO17 (Supplementary Table S4). The putative viral sequence of 11,682 nt was assembled from five Trinity contigs and had six predicted ORFs. The predicted L protein of this virus and that of AmNSRV1 shared ca. 28% identity, while the most closely related viruses according to Blastx and Blastp searches were Bondarzewia berkeleyi negative-strand RNA virus 1 (BbNSRV1)^34^ and Auricularia heimuer negative-stranded RNA virus 1 (AhNSRV1, GenBank accession MT259204, unpublished) (Table 1). AhNSRV1 is the proposed representative species of the new genus *Auriculariamonavirus* (2020.004F.Ac.v1.Mymona at https://talk.ictvonline.org/files/). The in silico detected virus sequence was named as Armillaria mellea negative strand RNA virus 2 (AmNSRV2, Table 1).

#### Putative members of family *Botourmiaviridae* in two South African isolates of *A. mellea*

**Figure 4:**
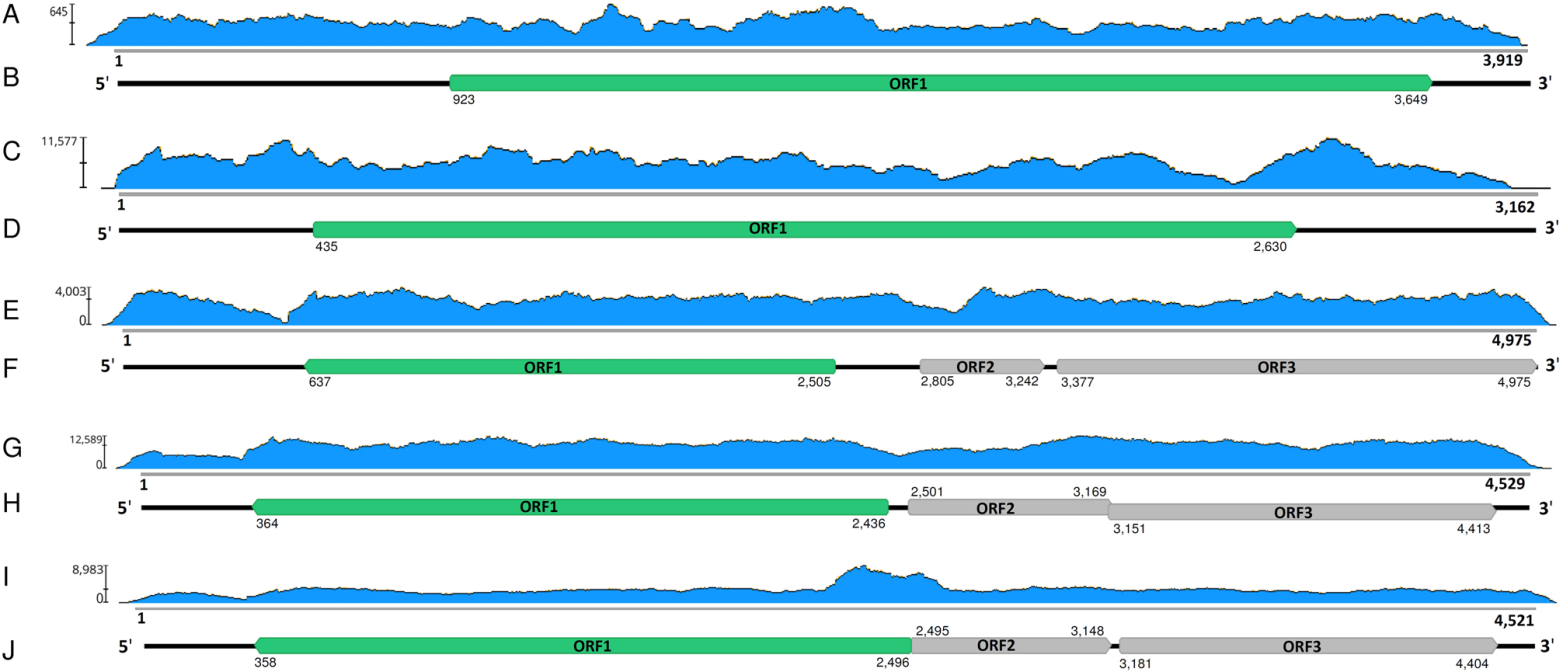
Genome organization of the Armillaria ourmia-like and ambi-like viruses. (**A**) Coverage of raw reads mapped against the Armillaria mellea ourmia-like virus 1 (AmOlV1) genome. (**B**) Schematic presentation of AmOlV1 genome with UTRs and predicted ORFs. (**C**) Coverage of raw reads mapped against the Armillaria mellea ourmia-like virus 2 (AmOlV2) genome. (**D**) Schematic presentation of AmOlV2 genome with UTRs and predicted ORFs. (**E**) Coverage of raw reads mapped against the Armillaria borealis ambi-like virus 1 (AbAlV1) genome hosted by isolate N40. (**F**) Schematic presentation of AbAlV1 genome with UTRs and predicted ORFs. (**G**) Coverage of raw reads mapped against the Armillaria borealis ambi-like virus 2 (AbAlV2) genome hosted by isolate N40. (**H**) Schematic presentation of AbAlV2 genome with UTRs and predicted ORFs. (**I**) Coverage of raw reads mapped against the Armillaria sp. ambi-like virus 3 (AsAlV3) genome hosted by isolate N40. (**J**) Schematic presentation of AsAlV3 genome with UTRs and predicted ORFs. Predicted translation initiation and termination sites are marked above or below each ORF. Predicted RdRPs coloured with green hue.

Two distinct ourmia-like contigs were detected in isolates of *A. mellea* (see Supplementary Table S5 online). Coding complete virus genome sequences were deposited in GenBank and named Armillaria mellea ourmia-like virus 1 (AmOlV1) and Armillaria mellea ourmia-like virus 2 (AmOlV2) (Table 1). The nonsegmented genomes of AmOlV1 (3,919 nt) and AmOlV2 (3,162 nt) contain only a single predicted ORF, encoding for a putative RdRP (Fig. 4A–D), and their partial UTRs were verified using Sanger sequencing. The predicted size of the RdRP in AmOlV1 and AmOlV2 was 908 aa (M_r_ = 103.450 kDa) and 731 aa (M_r_ = 81.976 kDa), respectively. Based on read mapping, there were seven and five polymorphic nucleotide sites in the coding region of AmOlV1 and AmOlV2 with variant frequencies of ca. 6–30%, respectively (see Supplementary Table S6 online).

**Figure 5:**
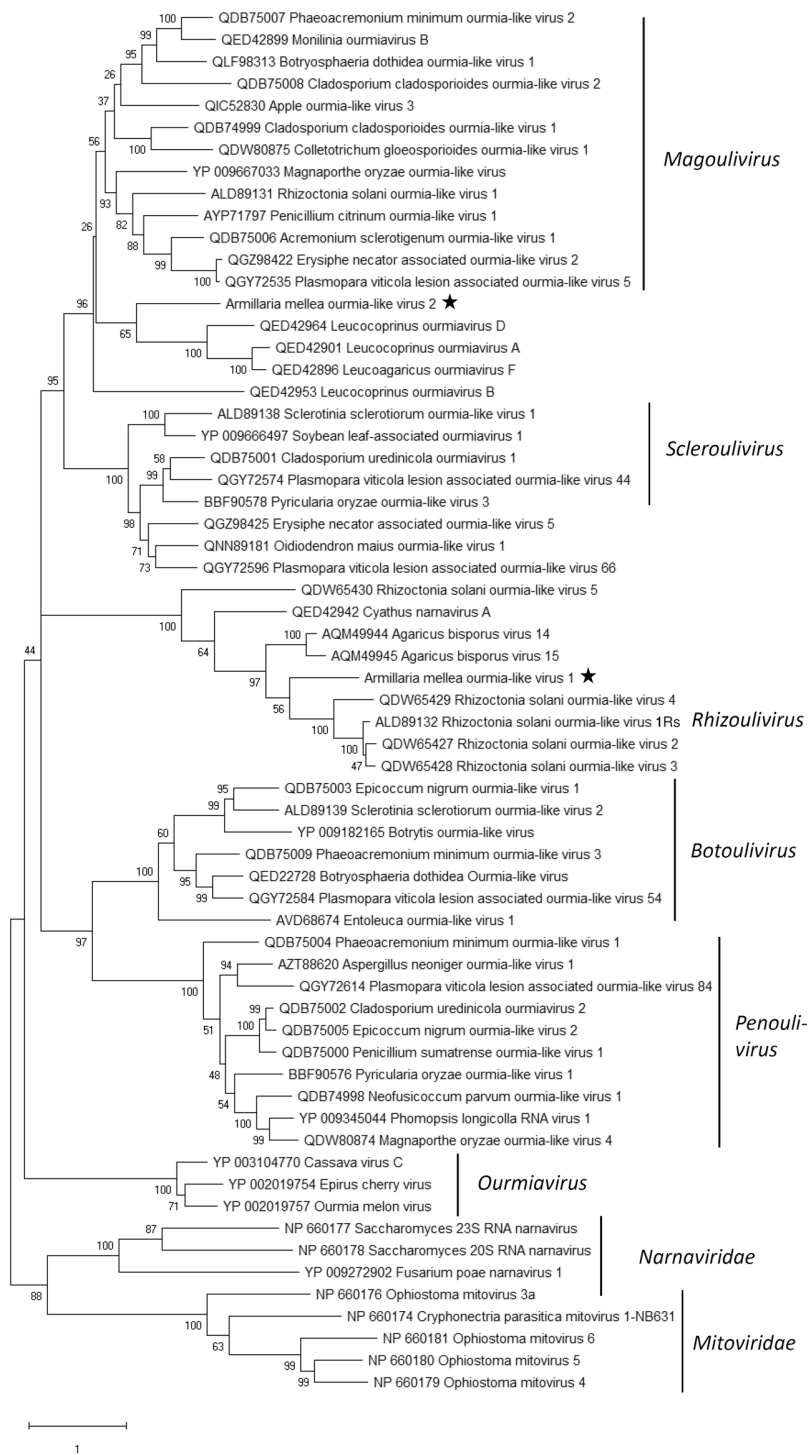
Phylogenetic tree of the family *Botourmiaviridae* and selected related families based on an alignment of RdRP aa sequences generated using MAFFT *v7.450* with Blosum62 substitution matrix. The evolutionary history was inferred by using the Maximum Likelihood method and Le_Gascuel_2008 model with 4 gamma categories (+ *G* + *I*). All positions with less than 95% site coverage were eliminated, i.e., fewer than 5% alignment gaps, missing data, and ambiguous bases were allowed at any position (partial deletion option). Evolutionary analyses were conducted in MEGA X with 1000 bootstrap repeats^86^. The *Armillaria* viruses are designated with a star.

AmOlV1 shared the highest Blastx identity with Agaricus bisporus virus 14^35^, and AmOlV2 with Apple ourmia-like virus 3, a magoulivirus in family *Botourmiaviridae* (GenBank accession QIC52830, unpublished) (Table 1). Phylogenetic analysis confirmed that AmOlV2 resembles members of genus *Magoulivirus,* whereas AmOlV1 clustered together with Rhizoctonia solani ourmia-like virus 1^36^, the proposed type species of new genus *Rhizoulivirus* and several unassigned ourmia-like viruses. Picarelli et al.^37^ suggested the existence of a separate family provisionally named “Basidionarnaviridae”, encompassing the Agaricus bisporus virus 15^35^ and Rhizoctonia ourmia-like viruses 2–5^37^. The divergent grouping of the Rhizoctonia ourmia-like viruses seems to be based on their long predicted ORF regions. However, delimiting the phylogenetic analysis to the commonly shared sequence region supported the grouping of these viruses together with the proposed genus *Rhizoulivirus* (Fig. 5). To our knowledge, the size of the polymerase has not been experimentally determined for these viruses.

### Viruses representing a new putative virus group called ambiviruses

**Figure 6:**
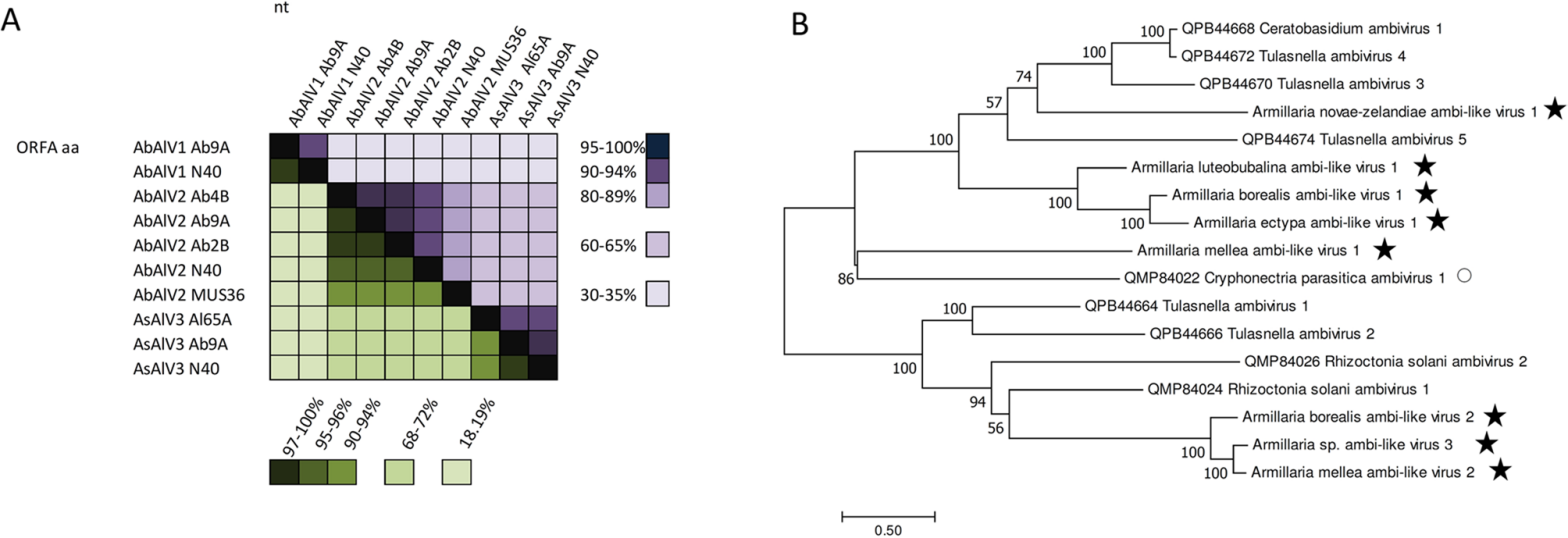
Pairwise identities of ambi-like viruses and phylogenetic analysis. (**A**) Armillaria borealis ambi-like virus 1 (AbAlV1), Armillaria borealis ambi-like virus 2 (AbAlV2) and Armillaria sp. ambi-like virus 3 (AsAlV3) hosted by *Armillaria borealis* isolates (Ab2B, Ab4B, Ab9A, MUS36 and N40) and *A. cepistipes* Al65A. Each cell presents pairwise identities between viral sequences. Alignments were generated with MAFFT in Geneious 10.2.6. Green hues represent pairwise identities between predicted RdRP aa sequences and violet hues those between complete nt sequences. (**B**) Phylogenetic tree of the ambi-virus like viruses was generated from predicted aa sequences of putative RdRPs aligned in MEGA7^88^ with MUSCLE and using the LG (+ F) model with G + I rates among sites (best-fit model according to ModelFinder) and 1000 bootstrap repetitions. All positions with less than 95% site coverage were eliminated, i.e., fewer than 5% alignment gaps, missing data, and ambiguous bases were allowed at any position. The *Armillaria* viruses are designated with a star. Cryphonectria parasitica ambivirus 1 is hosted by an ascomycetous host (indicated with open circle), all other ambi-like viruses have basidiomycetous hosts.

Three distinct ambi-like viruses were retrieved from our RNA-Seq library, and were all found to be hosted by more than one *Armillaria* isolate (Table 1, Fig. 6A). The complete sequences of ambi-like viruses 1–3 hosted by *A*. *borealis* isolate N40 comprised 4975, 4529 and 4521 nt, each encoding for three predicted ORFs (Table 1, Fig. 4E–J). None of the predicted proteins of the viruses showed conserved domains, but the longest predicted protein of each virus included the GDD motif, which is considered as the hallmark of RdRPs. Based on the nt and aa level sequence identities (Fig. 6A), the three ambi-like viruses hosted by N40 were not considered as variants of the same virus species (although no species delimitation criteria are available for this novel group of viruses^28,29^), and were designated as Armillaria borealis ambi-like virus 1 (AbAlV1), Armillaria borealis ambi-like virus 2 (AbAlV2) and Armillaria sp. ambi-like virus 3 (AsAlV3).

In addition, ambi-like viruses were discovered in five *Armillaria* RNA-Seq libraries deposited in the NCBI SRA database (Table 1; Supplementary Table S4; Supplementary Fig. S1; Supplementary Fig. S2). The raw reads were mapped against edited sequences of putative ambi-like viruses and were designated as Armillaria ectypa ambi-like virus 1, Armillaria luteobubalina ambi-like virus 1, Armillaria mellea ambi-like virus 1 (AmAlV1), Armillaria mellea ambi-like virus 2 (AmAlV2) and Armillaria novae-zelandiae ambi-like virus 1 (Table 1). AmAlV1 and AmAlV2 shared low sequence identities, 39.8% at the nt level and 23.8% at the aa level when the predicted translation of longest ORFs of both putative viruses were aligned using MAFFT (see Supplementary Fig. S2 online). The number of ambi-like virus genomes is currently very limited, and little is known about their occurrence and variability. However, based on a small-scale phylogenetic analysis the ambi-like viruses of *Armillaria* seem to be of polyphyletic origin as they were found in separate clusters including also ambiviruses from distantly related host fungi (Fig. 6B).

### Viral co-infections and interspecies divergence

All the other viruses except for the ambi-like viruses occurred in one host isolate only. However, some of them were found in mixed infections. For example, *A. borealis* N40 from Siberia hosted four different viruses, including the virga-like AbMV1 and three different ambi-like viruses. In addition, *A. mellea* CMW3973 harbored the mymona-like AmNSV1 and an ourmia-like virus (AmOlV2). The RNA-Seq library of *A. mellea* ELDO17 (BioProject PRJNA568830) analysed in silico hosted another mymona-like virus and an ambi-like virus. Only ambi-like viruses were found in the Finnish isolates, of which *A. borealis* Ab9A hosted all three ambi-like viruses. The occurrence of the different ambi-like viruses in a single host isolate further supports the notion that they represent distinct virus species. This is because mutual exclusion of conspecific virus strains is considered a species delimitation criterion in many viral taxa.

Sequence variants representing a single ambi-like virus (1, 2 or 3) hosted by different *A. borealis* isolates shared more than 90% nt sequence identities (Fig. 6A), even though variation was observed in the length and genome organization between virus variants (ORF structure; Supplementary Fig. S3). Moreover, AsAlV3 showed a higher sequence identity between variants from Finland (Ab9A) and Siberia (N40) from the same host species (*A. borealis*) than between Finnish variants Ab9A and Al65A from two different host species (*A. borealis* and *A. cepistipes*) (Fig. 6A).

### Phenotypes of virus-infected and virus-free *Armillaria* strains

In order to assess possible effects of viruses on the growth rate of their host, isogenic fungal strains with and without viral infections were generated using temperature treatment. Thermal treatment was successfully used to cure isolates N40, CMW3973, CMW50256, Al65A and Ab9A of viral infections. The highest incubation temperature used for the thermal treatment was 35 °C for isolates CMW50256 and Al65A and 36 °C for isolates N40, CMW3973 and Ab9A.

**Figure 7:**
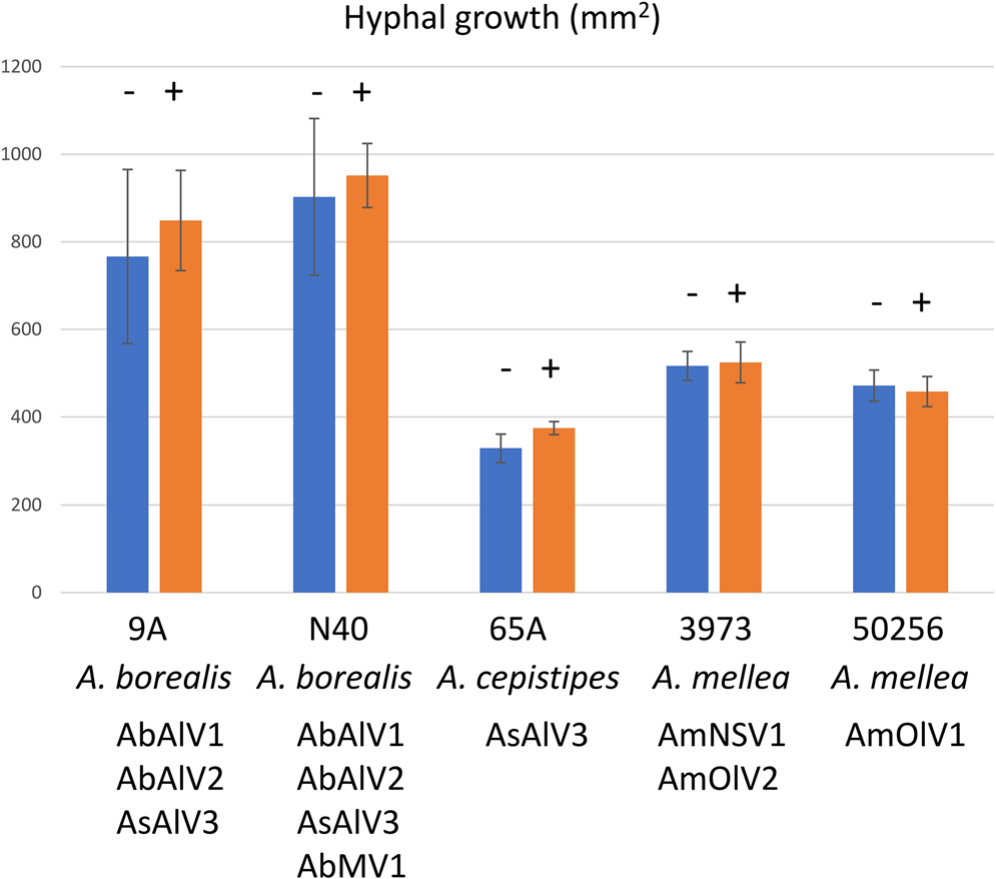
Growth of isogenic virus-infected and virus-free isolates of *Armillaria* in three weeks at 20 °C on 2% MEA agar plates. Blue (−) = thermal treated (virus-free); orange ( +) = virus-infected. The error bars indicate standard deviation. The t-test p-values ranged from 0.405 to 0.738 except for 65A that showed a p-value of 0.015 when comparing the isogenic strains (the growth of the virus-infected strain was on average 14% higher than that of the virus-free isogenic strain in three weeks). The number of replicate plates measured was 6 (4 for virus-free isolate N40 due to desiccation of two plates).

The growth rate of the original and isogenic heat-treated virus-free *Armillaria* strains was compared by recording their growth on 2% malt extract agar (MEA) plates during a three-week period (Fig. 7). The presence of viruses did not appear to have a major effect on the growth rate of the *Armillaria* isolates in laboratory conditions. In two cases, the hosts were originally infected with single viruses (an ambivirus or a botourmiavirus), whereas three hosts were cured of multiple virus infections (Fig. 7).

## Discussion

In this study, we describe the first genome sequences of mycoviruses hosted by the notorious, globally distributed plant pathogens of genus *Armillaria*. These new viruses belong to the families *Mymonaviridae*, *Botourmiaviridae* and *Virgaviridae* as well as to a recently described virus group tentatively named “ambiviruses”^28,29^. Many *Armillaria* species and other important root-rot fungi, such as *Heterobasidion* spp. (Bondarzewiaceae; Basidiomycota) and *Rosellinia necatrix* (Xylariaceae; Ascomycota), form large and long-living clonal individuals, which can be considered as optimal targets for viral biocontrol agents. Therefore, the viromes of both *Heterobasidion* spp. and *R. necatrix* have been extensively studied, which has led to the discovery of numerous mycoviruses, some of which cause host debilitation^21–23^. Interestingly, the persistent lifestyle of these root-rot pathogens seems to lead to the accumulation of viruses in the clonal individuals (via anastomosis contacts and spores)^38,39^ and even occasional virus transmission from other fungal species occurring in the same habitat^40–42^. In the case of *Armillaria* spp., there is no prior knowledge regarding the possible accumulation of viruses in ancient clones. Dvořák^43^ investigated the presence of dsRNA elements in 40 strains of *A. gallica, A. cepistipes* and *A. ostoyae* from the Czech Republic but none were detected. This is consistent with our findings that revealed only ssRNA viruses in the strains of *Armillaria* investigated in this study. Furthermore, only one negative-strand ssRNA virus was identified by in silico mining of transcriptomic datasets of several *Armillaria* species. Although more *Armillaria* isolates and species should be investigated for viral infections, our results showed that the virus diversity detected in *Armillaria* spp. is moderate compared to many other fungal species.

In this study, we identified a new virga-like virus in a Siberian strain of *A. borealis*. Classified members of family *Virgaviridae* infect plants and have rod-shaped, non-enveloped particles of about 20 nm in diameter and up to about 300 nm long^44^. However, virga-like viruses have been recently described in the basidiomycete *Auricularia heimuer*^32^, the ascomycetes *Macrophomina phaseolina*^36^ and *Podosphaera prunicola*^45^ and the oomycete *Plasmopara viticola* (Chromalveolata, Stramenopila)^46^. They have also been provisionally identified by metatranscriptomic analysis in the basidiomycetous *Leucocoprinus* sp., and the ascomycetous *Acidomyces richmondensis*^47^ and *Mycosphaerella* sp. (GenBank accession MK231048, unpublished). Members of family *Virgaviridae* are classified in seven genera, four of which include viruses transmitted by soilborne organisms: members of the genera *Furovirus, Pecluvirus, Pomovirus* are vectored by plasmodiophorids (Chromalveolata, Rhizaria, Cercozoa), for example *Polymyxa graminis* and *Spongospora subterranea*, whereas members of the genus *Tobravirus* are transmitted by nematodes^44^. The vast majority of virga-like viruses found in fungi are monopartite with a similar predicted ORF structure and genome organization as plant viruses of genus *Tobamovirus*, which are readily transmitted mechanically but lack known vectors. However, a tripartite virga-like virus related to unclassified invertebrate-derived viruses was recently discovered in *Aspergillus fumigatus*^48^. Notably, fungal virga-like viruses appear to encode a MP, a typical feature for plant viruses but usually lacking in mycoviruses. This supports the hypothesis that fungal viruses in the class *Alsuviricetes* could originate from plant viruses transmitted horizontally to plant-pathogenic fungi^49^. However, most mycoviruses in the class *Alsuviricetes* seem to have lost the MP and coat protein. In contrast to plant viruses, the persistent lifestyle and intracellular mode of transmission (via porous septa within a mycelium and anastomosis contacts between mycelia) generally enables mycoviruses to be transmitted without a MP, and the genes encoding such proteins may represent evolutionary remnants in the genomes of fungal virga-like viruses^50–52^.

Two isolates of *A. mellea* from South Africa also hosted positive-sense RNA viruses resembling members of family *Botourmiaviridae*. The evolutionary history of these positive-sense RNA viruses is intriguing and appears to follow an opposite trend compared to virgaviruses that seem to have been transmitted from plants to fungi. Thus, plant viruses in the genus *Ourmiavirus* have a genome segment encoding an RdRP resembling those of fungal narnaviruses, another segment encoding a MP related to of plant tombusviruses, and a third genome segment encoding a CP^27,53^. In contrast to the plant viruses, fungal ourmia-like viruses that are currently classified in four different genera have only one genome segment encoding the RdRP. Therefore, it appears that the plant ourmiaviruses have evolved from a narnavirus that has gained the proteins encoded by the two extra genome segments from plants to be able to move between plant cells^54^. Recently, a taxonomic proposal was submitted to accommodate two new genera and 25 new species in family *Botourmiaviridae* (2020.001F.A.v1.Botourmiaviridae; https://talk.ictvonline.org/files/). This suggests that this previously unknown virus group is highly common among fungi. The two ourmia-like viruses in *Armillaria* resemble viruses found in *Rhizoctonia solani* that are within the genus *Magoulivirus,* and proposed genus *Rhizoulivirus*, as well as several unclassified fungal and plant botourmiaviruses. Based on criteria for the classification of members of *Botourmiaviridae* by the ICTV^27^ and virus classification guidelines in the age of metagenomics^55^, coding complete sequences are sufficient for the classification of new virus species.

One isolate of *A. mellea* from South Africa hosted a negative-strand RNA virus related to members of family *Mymonaviridae.* In addition, another mymona-like virus was detected in silico from a North American strain of *A. mellea.* As expected, based on the origin of the host isolates, the two mymona-like viruses in *A. mellea* were relatively distantly related. *Mymonaviridae* is a relatively new family of negative-sense RNA viruses that have been found only in fungi^26^. They have enveloped filamentous particles of 25–50 nm in diameter and about 1000 nm in length. Mymonaviruses have been found in the fungal pathogens *Sclerotinia sclerotiorum* and *Fusarium graminearum,* and the basidiomycetous wood decay fungi *Lentinula edodes*^20^ and *Bondarzewia berkeleyi*^34^, as well as in metatranscriptomic datasets from soybean leaves and arthropods^36,56–60^. Currently there are nine classified species in two genera in family *Mymonaviridae* (2019.001F.R.Hubramonavirus_1gen at https://talk.ictvonline.org/files/). However, a recent proposal accepted by the ICTV Executive Committee (2020.004F.Ac.v1.Mymona) suggests the introduction of seven more genera in the family, including the genus *Lentimonavirus* where the newly characterized AmNSRV1 also groups based on phylogenetic analysis. AmNSRV2 was more closely associated with the genus *Auriculariamonavirus*. The elongated viral particles discovered earlier in North American strains of pathogenic *A. ostoyae* isolates seem to be at least 600–800 nm in length, and resemble those of mymonaviruses^31^.

It was notable that two *A. mellea* isolates from Cape Town were different in their viral composition, although both represent the same introduced pathogen of European origin. Strain CMW3973 isolated in 1996 from *Quercus robur* in the Company Gardens hosted a mymonavirus and a botourmiavirus, whereas isolate CMW50256 isolated in 2015 from *Leucadendron conocarpodendron* in the Kirstenbosch National Botanical Garden hosted another putative species of *Botourmiaviridae*. The two botourmiaviruses shared only ca. 20% aa sequence identity, therefore indicating that they are separate infections. Isolate CMW3973 represents *A. mellea* genotype A2 and isolate CMW50256 genotype A^9^, which are somatically compatible. It is considered that these closely related genotypes originate from a single introduction event of the pathogen into South Africa in the 1600s^8^.

Ambi-like viruses were found to be very common in isolates of *Armillaria* and occurred in almost all isolates from the Northern hemisphere. “Ambiviruses” constitute a new group of putative viruses that was recently identified by HTS in endomycorrhizal fungi^28^ as well as in *Cryphonectria parasitica* and *Rhizoctonia solani*^29^. Based on our ongoing investigations this virus group seems to be very common among asco- and basidiomycetes, but so far there is no information on their morphology, biology or population structure. The virus-like particles described by Blattný et al.^30^ in *A. mellea* strains from the Czech Republic were described as either rod-shaped (22–28 × 119 nm) or isometric (30 nm). As botourmiaviruses are capsidless, these two viral particle types suggest the presence of a yet uncharacterized virus group, but at this point we cannot verify whether one of them represents “ambiviruses”. Moreover, Blattný and colleagues considered their results preliminary as no negative staining was performed. Very similar viral strains occurred in *A. borealis* isolates from two different sites in Finland as well as Siberia, and only one *A. borealis* isolate (Ab3B) was free of ambivirus infections. All three ambivirus types co-infected the Siberian *A. borealis* isolate N40 and the Finnish *A. borealis* isolate Ab9A. Furthermore, AsAlV3 occurred in one strain of *A. cepistipes* in addition to these two *A. borealis* isolates. Interestingly, the sequence identity of the AsAlV3 strain from *A. cepistipes* and *A. borealis* from Finland was lower than that between the two *A. borealis* strains originating far from each other, suggesting that the ambivirus population might have differentiated based on its host species. However, further studies are needed to decipher whether the *Armillaria* viruses are species-specific. The Finnish *A. borealis* isolates infected with ambiviruses were all somatically incompatible and were therefore deemed to represent different clonal individuals, although their collection sites were relatively closely located in Southern Finland. Korhonen^61^ reported that the clone size of *A. borealis* (at that time designated as “species A”) was 120–150 m in diameter, and suggested their age to be about 100 years.

In this study, we were able to cure the *Armillaria* isolates from viruses and made a pilot growth experiment to investigate the possibility that viruses could affect the phenotype of their host. No major growth differences were observed between the virus-free and virus-infected isogenic strains, but it should be noted that the laboratory growth rate of virus-infected fungal strains is not necessarily reduced even if they have a lower virulence in natural conditions^62^. Moreover, mycovirus effects have been shown to be dependent on environmental and ecological conditions, such as temperature and fungal competitors present in the substrate^63,64^, and infections by multiple co-infecting viruses may have a different phenotypic outcome than single virus infections^65^. Future studies should determine whether viral infections are transmitted to *Armillaria* basidiospores and if they are present in actively growing hyphal tips. In this study, the virus-infected fungal isolates originated from fruiting body tissue (Finnish isolates), from wood (Siberian *A. borealis*) or from diploid mycelia from rhizomorphs, mycelial fans or *A. mellea* mycelium growing on the roots and root collars (South African isolates). This shows that the viruses are carried into different types of mycelia of *Armillaria*, and potentially have a possibility to spread into the mycelial network formed by large clonal individuals.

## Methods

### Origin and culturing conditions of the *Armillaria* isolates

A total of 18 *Armillaria* sp. strains were isolated from fruiting body tissue samples collected mostly from Southern Finland in September 2017 (see Supplementary Table S1 online). In addition, *A. borealis* strains from Siberia and *A. mellea* and *A. gallica* strains from South Africa were obtained from the culture collection (CMW) of Forestry and Agricultural Biotechnology Institute (FABI) and V.N. Sukachev Institute (Supplementary Table S1).

The species determination of the Finnish *Armillaria* strains was based on fruitbody morphology as well as molecular characterization of the Tef-1a gene region with primer pair EF595F/EF1160R or IGS rDNA with primer pair LR12R/O-1^66–68^. The *A. borealis* isolates originating from closely located sampling sites in Southern Finland (see Supplementary Table S1 online) were incubated as dual cultures to decipher whether they represented the same clonal individual based on somatic compatibility as described by Korhonen^61^. Approximate distance between the collection sites was as follows: 17 m between Ab3 and Ab4; 150 m between Ab2 and Ab9; 770 m between Ab3/Ab4 and Ab9; 780 m between Ab3/Ab4 and Ab2 (see Supplementary Table S1 for the CMW numbers). Isolates Ab3 and Ab4 were somatically compatible whereas the other isolates were not.

### Virus screening by cellulose chromatography

The *Armillaria* spp. culture collection was screened for dsRNA viruses using the cellulose chromatography method^69,70^. Briefly, the isolates were grown on liquid medium containing 2% malt and 1.5% yeast extract for 2–4 weeks, and ca. 2 g of mycelia were harvested and homogenized in a lysis buffer, followed by phenol and chloroform extractions and specific precipitation of dsRNA using Sigma cellulose fibres (medium) in 15% ethanol concentration. The presence and size of dsRNA was estimated by agarose gel electrophoresis. A virus-infected *Heterobasidion* isolate (*H. parviporum* 7R18 or RT3.49C hosting the partitivirus HetPV2-pa1 or HetPV4-pa1, respectively^71^ was included in each assay as a control. The isolates used for screening included 9 strains of *A. borealis* from Russia; 16 and 6 strains of *A. borealis* and *A. cepistipes* from Finland, and 29 strains of *A. mellea* and 3 strains of *A. gallica* from South Africa (see Supplementary Table S1 online).

### Total RNA extraction and HTS

For the extraction of total RNA, *Armillaria* mycelia was freeze-dried for 72 h prior to homogenization in liquid nitrogen. Total RNA was extracted using Spectrum Plant Total RNA Kit (Sigma-Aldrich) as described earlier^28^. RNA quality and quantity was determined using NanoDrop (Thermo Scientific) and by analysing an aliquot of each RNA by an agarose gel electrophoresis.

An RNA-Seq library was constructed using pooled RNA samples from nine *Armillaria* strains: N40, CMW3973, CMW50256, Ab2B, Ab3B, Ab4B, Ab9A, Al65A and MUS36. The RNA pool included 1 µg of total RNA from each fungal strain, except for strain N40 that was represented by two separate RNA extractions (2 µg). The RNA pool was sent in ethanol precipitation to Macrogen Korea for further quality determination, library construction and HTS. The library was generated using TruSeq Stranded Total RNA Library prep kit with Human/Mouse/Rat Gold for rRNA removal (Illumina) and 101 bp paired-end reads were obtained using Novaseq (Illumina).

### Bioinformatics and in silico mining of virus sequences from transcriptomic data

The raw reads were preprocessed with Trimmomatic^72^ and de novo assembly was conducted using Trinity (version 2.8.4 or 2.8.5)^73^ in R^74^. Host-specific contigs were determined with a Blastx run (e-value 10e-6) using a custom *Armillaria* protein database (db), generated by combining the *A*. *solidipes*, *A*. *gallica* and *A*. *ostoyae* protein models^75^. Contigs showing significant Blastx similarity with *Armillaria* protein models were omitted and a subsequent Blastx run (e-value 10e-3) was performed against a custom viral protein db. Contigs longer than 500 nt and resembling viral proteins were then re-examined using Blastn and Blastx searches against complete nucleotide and non-redundant protein sequences. Geneious 10.2.6 (Biomatters Ltd) was used for further analysis of the putative virus contigs and mapping of reads. Additionally, fifteen publicly available RNA-Seq libraries representing 13 different *Armillaria* species (see Supplementary Table S4 online) were analysed for the presence of RNA viruses using the same bioinformatics pipeline. The genome drafts of the *Armillaria* strains hosting putative mycoviruses were analysed with Blastn to confirm the putative viruses were not detected in the host DNA (see Supplementary Table S8 online).

In order to determine putative ORFans in the *Armillaria* library, Trinity contigs (not showing similarity with *Armillaria* or putative viral contigs) ≥ 1500 nt in length, having ORF ≥ 400 nt were selected and examined with Blastn, Blastp and Blastx searches against complete nucleotide and non-redundant protein sequences. Finally, the raw reads of the *Armillaria* library were mapped against contigs with no hits (see Supplementary Table S3 online).

### Validation of virus-like contigs

A 2-µg sample of each total RNA included in the RNA pool was converted to cDNA using RevertAid Reverse Trancriptase (Thermo Fisher Scientific) and random hexamer primers. Determination of host strains was performed using RT-PCR with DreamTaq DNA Polymerase (Thermo Fisher Scientific) and virus-specific primers (see Supplementary Table S9 online).

To investigate the possibility of genomic integration of the viral sequences, two nucleic acid extractions were performed for *Armillaria* strains N40, MUS36, CMW3973, CMW50256, and Al65A. Total nucleic acids were extracted using a modified phenol–chloroform extraction method described by Vainio et al.^76^ and DNA using the E.Z.N.A Forensic DNA Kit (Omega Bio-tek). Diluted nucleic acid samples were used as templates in standard PCR with DreamTaq DNA Polymerase and virus-specific primers (the same as used for host screening). The quality of DNA was verified by using it as a template for PCR amplification of ITS rDNA and M13 minisatellite markers^77^ as described earlier^78^. Putative virus contigs DN471, DN562 and DN2538 were confirmed to occur in more than one *Armillaria* strain. For contig DN471 genomic integration was tested using DNA of N40. DNA of N40 and DNA of MUS36 were used in testing the integration of contig DN562 and DNA of N40 and DNA of Al65A for DN2538.

### Determination of complete virus genome sequences

The single primer amplification technique of Lambden et al.^79^ was used for attempts to determine the viral 5′ and 3′ UTRs. The T4 adapter or primer A blocked at the 3´end by an NH_2_ group^80,81^ was ligated to viral dsRNA or total RNA using the T4 RNA ligase (Thermo Fisher Scientific), followed by purification of RNA by agarose gel electrophoresis and extraction using the RNAid kit (Bio101, Carlsbad) and reverse transcription. Initial denaturation was conducted for 3–6 min at 99 °C, and reverse transcription (1 h 30 min) at 55 °C, 60 °C and 50 °C for Maxima H Minus Reverse Transcriptase (Thermo Fisher Scientific), SuperScript IV (Invitrogen) and RevertAid H minus Reverse Transcriptase (Thermo Fisher Scientific), respectively. Phusion High-Fidelity DNA Polymerase (Thermo Fisher Scientific) was used for PCRs together with a primer complementary to the T4 RNA adapter or primer A and a virus specific primer (Supplementary Table S9). Each sequence position was characterized by analysing at least two Sanger sequences determined at Macrogen Europe. The complete sequences of putative viral contigs showing similarity to genomes of ambiviruses were determined from all *A. borealis* and *A*. *cepistipes* hosts (Table 1) using Phusion High-Fidelity DNA Polymerase and virus specific primers (see Supplementary Table S9 online).

### Virus curing and growth rate measurements

*Armillaria* strains N40, CMW3973, CMW50256, Al65A and Ab9A were grown on 2% MEA plates at RT prior to the initiation of thermal treatments. The plates were first incubated for a week at 32 °C in the dark where after the temperature was risen gradually every week up to 38 °C until the mycelial growth halted. The growth of the cultures was investigated weekly and after the final incubation, the fungal cultures were recovered on 2% MEA plates in RT for 9 days. RNA was extracted from mycelia with Spectrum Plant Total RNA Kit and the presence of mycoviruses was analysed with RT-PCR as described above. In order to validate the cDNA synthesis, the same templates were used for the amplification of host-derived marker molecules. The primer pair ITS1-F/ITS4 was used for the amplification of ITS rDNA^82,83^, and the primer pair for EF595F/EF1160R the amplification of Tef-1a. The total RNA extracts used as templates were treated with DNase I (Thermo) to prevent amplification from DNA templates. The same RNA extract without DNase treatment yielded ITS rDNA/Tef-1a amplification products, whereas the DNase-treated template without reverse transcription did not, hence validating the functionality of the DNase treatment.

The growth rates of isogenic virus infected and cured (virus-free) *Armillaria* strains were compared by inoculating each fungal strain on 2% MEA plates and recording their growth during three weeks at room temperature (20 °C) in the dark. The inoculum was an agar piece of 0.5 mm in diameter from the actively growing part of the mycelium. There were six replicate cultures for each fungal strain. The ImageJ program of the Fiji Platform^84^ was used to measure the area of mycelial growth, and the statistical difference between isogenic virus-infected and virus-free fungal strains was tested using t-test in Microsoft Excel 2016 (two sample assuming unequal variances).

## Supplementary Information

Supplementary Information 1.

Supplementary Information 2.

Supplementary Information 3.

Supplementary Information 4.

Supplementary Information 5.

Supplementary Information 6.

## Data Availability

Nucleotide sequence data reported are available in the GenBank database under the accession numbers MW423800-13 and TPA: BK014417-22, and the RNA-Seq reads are deposited in the SRA and can be accessed through PRJNA685612. The datasets generated during and/or analysed during the current study are available in the NCBI SRA repository under the BioProject numbers listed in Table 1.
