# Supplementary material for: Armillaria root rot fungi host single-stranded RNA viruses
Source: Sci Rep. 2021 Apr 1;11:7336. doi: 10.1038/s41598-021-86343-7 (PMC8016926; doi:10.1038/s41598-021-86343-7)
Supplement: Supplementary file 2 — Supplementary Information 2. [file 41598_2021_86343_MOESM2_ESM.docx]

Supplementary Information for

”*Armillaria* root rot fungi host single-stranded RNA viruses”

By Linnakoski et al. (2021)

This PDF includes the following data:

Supplementary Table S1. *Armillaria* isolates used in this study

Supplementary Table S3. ORFan contigs detected in our *Armillaria* RNA-Seq library

Supplementary Table S4. *In silico* analysed *Armillaria* sp. RNA-Seq libraries

Supplementary Table S5. Reads mapped to putative viral Trinity contigs of *Armillaria* RNA-Seq libraries

Supplementary Table S6. Variant calling for Armillaria mellea ourmia-like virus 1 (AmOlV1), Armillaria mellea ourmia-like virus 2 (AmOlV2), Armillaria borealis mycovirgavirus 1 (AbMV1) and Armillaria mellea negative strand RNA virus 1 (AmNSRV1)

Supplementary Table S7. Armillaria borealis ambi-like virus 1, Armillaria borealis ambi-like virus 2 and Armillaria sp. ambi-like virus 3 hosted by *A. borealis* (Ab2B, Ab4B, Ab9A, MUS36) and *A. cepistipes* (Al65A) isolates

Supplementary Table S8. Primers utilized in the current study

Supplementary Table S9. The BioProject and SRA IDs (NCBI) utilized in Blastn analyses conducted in order to determine the possibility of virus integration to host genome

Supplementary Fig. S1. Genome organization of the ambi-like viruses retrieved from *Armillaria* RNAseq libraries *in silico*.

Supplementary Fig. S2. Pairwise identities of ambi-like viruses hosted by *Armillaria* sp.

Supplementary Fig. S3. Genome organization of the ambi-like viruses hosted by *Armillaria borealis* and *A. cepistipes.*

Supplementary References

**Supplementary Table S1.** *Armillaria* isolates used in this study

| **Species** | **Isolate no.** | **Culture collection no^1^** | **Host** | **Substrate** | **Location** | **Country** | **Date collected** | **Collector** |
| --- | --- | --- | --- | --- | --- | --- | --- | --- |
| *A. borealis* | Ab1A | 52573 | *Betula* sp. stump | Fruiting body | Männistönmuorinpolku, Mankkaa, Espoo | Finland | 17.9.2017 | E. Vainio |
|  | Ab2B | 52574 | *Betula* sp. stump | Fruiting body | Kannaksenpuisto, Pohjois-Tapiola, Espoo | Finland | 17.9.2017 | E. Vainio |
|  | Ab3A | 52575 |  | Fruiting body | Mankkaanpuronnotko, Mankkaa, Espoo | Finland | 17.9.2017 | E. Vainio |
|  | Ab3B |  |  | Fruiting body | Mankkaanpuronnotko, Mankkaa, Espoo | Finland | 17.9.2017 | E. Vainio |
|  | Ab4A | 52576 |  | Fruiting body | Mankkaanpuronnotko, Mankkaa, Espoo | Finland | 17.9.2017 | E. Vainio |
|  | Ab4B |  |  | Fruiting body | Mankkaanpuronnotko, Mankkaa, Espoo | Finland | 17.9.2017 | E. Vainio |
|  | Ab5A | 52577 |  | Fruiting body | Kiiltokallionreuna, Espoo | Finland | 17.9.2017 | E. Vainio |
|  | Ab6A | 52578 |  | Fruiting body | Kannaksenpuisto, Pohjois-Tapiola, Espoo | Finland | 17.9.2017 | E. Vainio |
|  | Ab7A | 52580 |  | Fruiting body | Mankkaanhaka, Espoo | Finland | 17.9.2017 | E. Vainio |
|  | Ab8A | 52581 | Hardwood tree | Fruiting body | Kiiltokallionreuna, Espoo | Finland | 17.9.2017 | E. Vainio |
|  | Ab9A | 52582 |  | Fruiting body | Kannaksenpuisto, Pohjois-Tapiola, Espoo | Finland | 17.9.2017 | E. Vainio |
|  | Ab10A | 52583 |  | Fruiting body | Kannaksenpuisto, Pohjois-Tapiola, Espoo | Finland | 17.9.2017 | E. Vainio |
|  | Ab13A | 52586 |  | Fruiting body | Niskala, Helsinki | Finland | 17.9.2017 | E. Vainio |
|  | Ab14A | 52587 |  | Fruiting body | Niskala. Helsinki | Finland | 17.9.2017 | E. Vainio |
|  | MUS36 | 52602 | *Larix* sp. | Fruiting body | Koivikon Kievari, Kitee | Finland | 5.10.2017 | P. Veteli |
|  | MUS37 | 52589 | *Betula* sp. | Fruiting body | Hopealahti, Joensuu | Finland | 21.9.2017 | P. Veteli |
|  | D11 |  | *Betula pendula* | Wood | Krasnoyarsk, Siberia | Russia | 25.9.2014 | I. Pavlov |
|  | M30 |  | *Populus tremula* | Mycelium fan | Krasnoyarsk, Siberia | Russia | 1.10.2014 | Y. Litovka |
|  | N31 |  | *Populus tremula* | Wood | Krasnoyarsk, Siberia | Russia | 1.10.2014 | I. Pavlov |
|  | N35 |  | *Populus tremula* | Wood | Krasnoyarsk, Siberia | Russia | 1.10.2014 | I. Pavlov |
|  | N36 |  | *Populus tremula* | Wood | Krasnoyarsk, Siberia | Russia | 1.10.2014 | Y. Litovka |
|  | N39 |  | *Populus tremula* | Wood | Krasnoyarsk, Siberia | Russia | 30.9.2014 | Y. Litovka |
|  | N40 |  | *Populus tremula* | Wood | Krasnoyarsk, Siberia | Russia | 30.9.2014 | I. Pavlov |
|  | N43 |  | *Populus tremula* | Wood | Krasnoyarsk, Siberia | Russia | 30.9.2014 | I. Pavlov |
|  | D48 |  | *Populus tremula* | Wood | Krasnoyarsk, Siberia | Russia | 16.10.2014 | Y. Litovka |
| *A. cepistipes* | Al65A | 52591 |  | Fruiting body | Ruotsinkylä, Tuusula | Finland | 23.9.2017 | E. Vainio |
|  | Al100A | 52596 |  | Fruiting body | Villa Elfvik, Espoo | Finland | 29.9.2017 | E. Vainio |
|  | Al101A | 52597 | *Picea abies* | Fruiting body | Lukukallio forest, Mankkaa, Espoo | Finland | 30.9.2017 | E. Vainio |
|  | Al102 | 52599 | *Betula* sp. | Fruiting body | Lukukallio forest, Mankkaa, Espoo | Finland | 30.9.2017 | E. Vainio |
|  | MUS34 | 52601 | *Alnus* sp. | Fruting body | Lammi | Finland | 29.9.2017 | P. Veteli |
|  | MUS35 |  | *Sorbus* sp. | Fruiting body | Lammi | Finland | 29.9.2017 | P. Veteli |
| *A. gallica* |  | 49613 | *Olea capensis subsp. capensis* | Root | Kirstenbosch National Botanical Garden, Cape Town | South Africa | 14.10.2014 | M.P.A. Coetzee |
|  |  | 49617 | *Olea capensis* | Root | Kirstenbosch National Botanical Garden, Cape Town | South Africa | 15.1.2015 | J. Roux |
|  |  | 49619 | *Virgilia oroboides* | Root | Kirstenbosch National Botanical Garden, Cape Town | South Africa | 15.1.2015 | J. Roux |
| *A. mellea* |  | 3341 | *Quercus* sp. |  | Cape Town | South Africa | 1.6.1997 | M.P.A. Coetzee |
|  |  | 3787 | *Hydrangium* sp. |  | Cape Town | South Africa | 1.6.1997 | M.P.A. Coetzee |
|  |  | 3973 | *Quercus robur* |  | Cape Town | South Africa | 1.2.1996 | M.J. Wingfield |
|  |  | 3974 | *Quercus robur* |  | Cape Town | South Africa | 1.2.1996 | M.J. Wingfield |
|  |  | 3975 | *Quercus spp* |  | Cape Town | South Africa | 1.2.1996 | M.J. Wingfield |
|  |  | 3976 | *Quercus* sp. |  | Cape Town | South Africa | 1.2.1996 | M.J. Wingfield |
|  |  | 3977 | *Quercus* sp. |  | Cape Town | South Africa | 1.2.1996 | M.J. Wingfield |
|  |  | 3979 | *Quercus robur* |  | Cape Town | South Africa | 1.2.1996 | M.J. Wingfield |
|  |  | 3981 | *Quercus robur* |  | Cape Town | South Africa | 1.2.1996 | M.J. Wingfield |
|  |  | 4302 | *Quercus robur* |  | Cape Town | South Africa | 1.6.1997 | Coetzee MPA |
|  |  | 4303 |  |  | Cape Town | South Africa | 1.6.1997 | Coetzee MPA |
|  |  | 4304 |  |  | Cape Town | South Africa | 1.6.1997 | M.P.A. Coetzee |
|  |  | 4305 | *Quercus robur* |  | Cape Town | South Africa | 1.6.1997 | M.P.A. Coetzee |
|  |  | 4306 | *Quercus robur* |  | Cape Town | South Africa | 1.6.1997 | M.P.A. Coetzee |
|  |  | 4307 | *Hydrangium* sp. |  | Cape Town | South Africa | 1.6.1997 | M.P.A. Coetzee |
|  |  | 49610 | *Leucadendron strobilinum* | Root | Kirstenbosch National Botanical Garden, Cape Town | South Africa | 14.10.2014 | M.P.A. Coetzee |
|  |  | 49611 | *Leucadendron macowanii* | Root | Kirstenbosch National Botanical Garden, Cape Town | South Africa | 14.10.2014 | M.P.A. Coetzee |
|  |  | 49612 | *Leucospermum conocarpodendron* | Root | Kirstenbosch National Botanical Garden, Cape Town | South Africa | 14.10.2014 | M.P.A. Coetzee |
|  |  | 49620 | *Widdringtonia schwarzii* | Root | Kirstenbosch National Botanical Garden, Cape Town | South Africa | 15.1.2015 | J. Roux |
|  |  | 49621 | *Widdringtonia schwarzii* | Root | Kirstenbosch National Botanical Garden, Cape Town | South Africa | 15.1.2015 | J. Roux |
|  |  | 49622 | *Leucadendron argenteum* | root | Kirstenbosch National Botanical Garden, Cape Town | South Africa | 15.1.2015 | J. Roux |
|  |  | 49628 | *Cliffortia ferruginea* | Root | Table Mountain, Cape Town | South Africa | 15.10.2014 | M.P.A. Coetzee |
|  |  | 49629 | *Virgilia oroboides* | Root | Table Mountain, Cape Town | South Africa | 15.10.2014 | M.P.A. Coetzee |
|  |  | 49630 | *Virgilia oroboides* | Root | Table Mountain, Cape Town | South Africa | 15.10.2014 | M.P.A. Coetzee |
|  |  | 49631 | *Virgilia oroboides* | Root | Table Mountain, Cape Town | South Africa | 15.10.2014 | M.P.A. Coetzee |
|  |  | 49632 | *Virgilia oroboides* | Root | Table Mountain, Cape Town | South Africa | 15.10.2014 | M.P.A. Coetzee |
|  |  | 49633 | *Virgilia oroboides* | Root | Table Mountain, Cape Town | South Africa | 14.10.2014 | M.P.A. Coetzee |
|  |  | 50256 | *Leucospermum conocarpodendron* | Root | Kirstenbosch National Botanical Garden, Cape Town | South Africa | 15.10.2014 | M.P.A. Coetzee |
|  |  | 50257 | *Leucadendron argenteum* | Root | Kirstenbosch National Botanical Garden, Cape Town | South Africa | 15.1.2015 | J. Roux |

^1^CMW: Culture Collection of the Forestry and Agricultural Biotechnology Institute (FABI), University of Pretoria, Pretoria, South Africa.

**Supplementary Table S3.** ORFan contigs detected in our *Armillaria* RNA-Seq library

| **Trinity Contig^a^** | **ORFs** | **predicted aa of ORF** | **Mapping reads^b^** | **Average depth^c^** | **Contig lenght** | **Sequence** |
| --- | --- | --- | --- | --- | --- | --- |
| TRINITY_DN11424_c0_g1_i1 | 1 Partial | ALPTTTILHDLTPITTMPVALLKSNNLPTATLSLTTMLILPSEQVFGRPASILDQITELKVISQQTITTNRLRQKEEERAQTLRQELPSLYRCLEYWEARAAKKRKASSSLDVRPEDSHCPTSPIKRRRLDESPVTSTISPCTTGAIASIPCRIPRRIKRPDCSSLPSRPACKASKKPAKNGSKFAVEAKQPTKLLPVGAPPKRHASAQAKNTIRCNKLPAGAKPHAASVASGYSHMRDIRPATIEQRRRPWR | 2.044 | 193 | 1,600 | CACTGATGGAAGTTAGAGCGATGTGTTAATGGGAAAGGAAAGTTGATATGAAACTGCCAGTCTCTGGAGGACGCGCTGATCGTGAACCGACGGTAGACGTTGCACCAGAGTTCGTCGTCTATGATCCCAGAAAAATAGGGCGACGCGAGCGAAATGTATATAAGATCTTGCGGACTGCAGAATTCTAGAATCGTCATCATGAGCGAGGGATTGCGAGAAACTTCGAAGGCCCGAGCATGCTCACACTTGTCTAGTCGGATTTTCAGCGAAAGAAGAAGAGGACCGAGTTATACATGGGAGAACAATTTGAACAAGACGAGACCGAGAGGCGCCAAGTCATGTTATCAGACCAAATTTAGATCAAGACGACGCTGGCCTGAGCCTTTTCCAGAAACGGTATTGCACAATCGACGACAGTGTTAGACAGGGAAAACAACAAGATGCAACAGATTTGAGTATTGTAGCACTGGCACGGGGCAGAGTATAATCATAGTCTGAGTGAATGAACGCCGGAAGAATCGGCGTTGATGCGAAAGTGCCTAAATTTGAGATACCTTTTCATTGATATTTGTTAATCTGCGTATTTTGTTTATCATCTTGATGGATATTTGAGGATTAGGACAGGCAAAGGCTCGTCGTCGTTATTTTACACAAATTTATGAACTTAACACCTACAATGTCAAGCGACAAGGGAGTGAATGTCACTGTTCCTCCCGGCTTACTCCAAAAGAAGGTATATTCCCGATCCCCAAAACAAAGCCCTGATGATGATGTATAACAGTGTATAACCAATGAAGCCCACCAAATAAAACCCAAAGTCAAGAAATCGATCCGCTCTTCACCGCCATGGACGCCGCCTCTGTTCGATGGTGGCAGGCCTTATATCCCGCATATGGCTATAGCCAGATGCGACGGAAGCTGCATGGGGCTTTGCTCCTGCTGGTAGCTTGTTACACCTGATGGTGTTTTTTGCTTGTGCGGAGGCATGCCGTTTGGGTGGAGCACCAACTGGGAGAAGTTTCGTGGGCTGCTTGGCCTCTACGGCAAACTTGCTGCCATTCTTGGCAGGCTTCTTGGAGGCCTTACAGGCTGGGCGAGACGGGAGGCTAGAGCAGTCAGGACGCTTGATCCGGCGTGGAATCCGACAGGGTATGCTGGCTATGGCACCAGTAGTGCAAGGCGAGATTGTGCTGGTGACCGGGCTCTCATCCAGTCGCCGCCGCTTGATAGGGGAAGTTGGACAGTGGGAATCCTCCGGACGAACGTCCAAAGACGAGGACGCCTTCCTCTTCTTTGCTGCCCGTGCCTCCCAATACTCCAGGCACCTATAGAGCGATGGGAGCTCCTGGCGAAGAGTTTGCGCTCTTTCTTCTTCTTTTTGCCTTAGACGGTTGGTCGTAATCGTCTGTTGACTGATCACCTTGAGCTCGGTAATCTGATCAAGGATAGAGGCTGGACGGCCGAAGACTTGTTCCGACGGTAAGATAAGCATCGTCGTCAAGGACAGTGTAGCTGTGGGTAGGTTGTTGGACTTTAAAAGAGCGACGGGCATTGTAGTGATAGGCGTGAGGTCGTGAAGGATCGTGGTAGTTGGTAGAGC |
| TRINITY_DN11424_c0_g1_i2 | 1 Partial | ALPTTTILHDLTPITTMPVALLKSNNLPTATLSLTTMLILPSEQVFGRPASILDQITELKVISQQTITTNRLRQKEEERAQTLRQELPSLYRCLEYWEARAAKKRKASSSLDVRPEDSHCPTSPIKRRRLDESPVTSTISPCTTGAIASIPCRIPRRIKRPDCSSLPSRPACKASKKPAKNGSKFAVEAKQPTKLLPVGAPPKRHASAQAKNTIRCNKLPAGAKPHAASVASGYSESAYLSTSCLTDHTFRPYAGYKACHHRTEAASMAVKSGSIS | 2,866 | 176.2 | 1,649 | CACTGATGGAAGTTAGAGCGATGTGTTAATGGGAAAGGAAAGTTGATATGAAACTGCCAGTCTCTGGAGGACGCGCTGATCGTGAACCGACGGTAGACGTTGCACCAGAGTTCGTCGTCTATGATCCCAGAAAAATAGGGCGACGCGAGCGAAATGTATATAAGATCTTGCGGACTGCAGAATTCTAGAATCGTCATCATGAGCGAGGGATTGCGAGAAACTTCGAAGGCCCGAGCATGCTCACACTTGTCTAGTCGGATTTTCAGCGAAAGAAGAAGAGGACCGAGTTATACATGGGAGAACAATTTGAACAAGACGAGACCGAGAGGCGCCAAGTCATGTTATCAGACCAAATTTAGATCAAGACGACGCTGGCCTGAGCCTTTTCCAGAAACGGTATTGCACAATCGACGACAGTGTTAGACAGGGAAAACAACAAGATGCAACAGATTTGAGTATTGTAGCACTGGCACGGGGCAGAGTATAATCATAGTCTGAGTGAATGAACGCCGGAAGAATCGGCGTTGATGCGAAAGTGCCTAAATTTGAGATACCTTTTCATTGATATTTGTTAATCTGCGTATTTTGTTTATCATCTTGATGGATATTTGAGGATTAGGACAGGCAAAGGCTCGTCGTCGTTATTTTACACAAATTTATGAACTTAACACCTACAATGTCAAGCGACAAGGGAGTGAATGTCACTGTTCCTCCCGGCTTACTCCAAAAGAAGGTATATTCCCGATCCCCAAAACAAAGCCCTGATGATGATGTATAACAGTGTATAACCAATGAAGCCCACCAAATAAAACCCAAAGTCAAGAAATCGATCCGCTCTTCACCGCCATGGACGCCGCCTCTGTTCGATGGTGGCAGGCCTTATATCCCGCATATGGCCTGAACGTGTGGTCTGTCAAACAAGAAGTAGATAGATAAGCAGACTCACTATAGCCAGATGCGACGGAAGCTGCATGGGGCTTTGCTCCTGCTGGTAGCTTGTTACACCTGATGGTGTTTTTTGCTTGTGCGGAGGCATGCCGTTTGGGTGGAGCACCAACTGGGAGAAGTTTCGTGGGCTGCTTGGCCTCTACGGCAAACTTGCTGCCATTCTTGGCAGGCTTCTTGGAGGCCTTACAGGCTGGGCGAGACGGGAGGCTAGAGCAGTCAGGACGCTTGATCCGGCGTGGAATCCGACAGGGTATGCTGGCTATGGCACCAGTAGTGCAAGGCGAGATTGTGCTGGTGACCGGGCTCTCATCCAGTCGCCGCCGCTTGATAGGGGAAGTTGGACAGTGGGAATCCTCCGGACGAACGTCCAAAGACGAGGACGCCTTCCTCTTCTTTGCTGCCCGTGCCTCCCAATACTCCAGGCACCTATAGAGCGATGGGAGCTCCTGGCGAAGAGTTTGCGCTCTTTCTTCTTCTTTTTGCCTTAGACGGTTGGTCGTAATCGTCTGTTGACTGATCACCTTGAGCTCGGTAATCTGATCAAGGATAGAGGCTGGACGGCCGAAGACTTGTTCCGACGGTAAGATAAGCATCGTCGTCAAGGACAGTGTAGCTGTGGGTAGGTTGTTGGACTTTAAAAGAGCGACGGGCATTGTAGTGATAGGCGTGAGGTCGTGAAGGATCGTGGTAGTTGGTAGAGC |

^a^Contigs not showing similarity with *Armillaria* or virus proteins having length more than 1500 nt and ORF more than 400 nt were selected, filtered by omitting contaminant contigs using Blastp with predicted proteins of *Paenibacillus polymyxa* CF05 (Lei et al. 2015) and analyzed with Blastn, Blastx and Blastp

^b^raw reads were mapped against the contig using Geneious for RNA Seq assembler with medium-low sensitivity

^c^mean value generated by Geneious 10.2.6

**Supplementary Table S4.** *In silico* analysed *Armillaria* sp. RNA-Seq libraries

| **BioProject** | **SRA run** | **Isolate** | **Reference** | ***In silico* detected mycoviruses** |
| --- | --- | --- | --- | --- |
| PRJNA519844 | SRR8536931 | *Armillaria borealis* FPL87.14 | JGI, Francis M. Martin^a^ | ND |
| PRJNA629806 | SRR11680630 | *Armillaria cepistipes* B12 | Sahu et al. (2020) | ND |
| PRJNA455898 | SRR7968262 | *Armillaria ectypa* FPL83.16 | JGI, Francis M. Martin^a^ | ambi-like virus |
| PRJNA677811 | SRR13091223 | *Armillaria fumosa* CBS 122221 | JGI, Francis M. Martin^a^ | ND |
| PRJNA633976 | SRR11816226 | *Armillaria gallica* 012m | Zhan et al. (2020) | ND |
| PRJNA455055 | SRR7777675 | *Armillaria luteobubalina* HWK02 | JGI, Jonathan M. Plett^a^ | ambi-like virus |
| PRJNA297618 | SRR2545913 | *Armillaria mellea* | Tsai et al. (2016) | ambi-like virus |
| PRJNA568830 | SRR10392772 | *Armillaria mellea* ELDO17 | JGI, Francis M. Martin^a^ | mymona-like virus and ambi-like virus |
| PRJNA500235 | SRR8442071 | *Armillaria nabsnona* CMW6904 | JGI, Francis M. Martin^a^ | ND |
| PRJNA677795 | SRR13091473 | *Armillaria novae-zelandiae* 2840 | JGI, Francis M. Martin^a^ | ambi-like virus |
| PRJNA629791 | SRR11680504 | *Armillaria ostoyae* strain: C18 | Sahu et al. (2020) | ND |
| PRJNA565538 | SRR10120337 | *Armillaria sinapina* strain XL17_48 | Fradj et al. (2020) | ND |
| PRJNA519845 | SRR8536922 | *Armillaria tabescens* CCBAS 213 | JGI, Francis M. Martin^a^ | ND |

^a^contact person for unpublished datasets of JGI (Joint Genome Institute)

ND: not detected

**Supplementary Table S5.** Reads mapped to putative viral Trinity contigs of *Armillaria* RNA-Seq libraries

| **BioProject/SRA ID** | **Mycovirus** | **Trinity contig (longest)** | **ORFs** | **Mapping reads^a^** | **Mean depth^b^** | **Contig length (nt)** | **Accession with the highest identity^c^** | **e-value** | **percent identity** | **query cover %** |
| --- | --- | --- | --- | --- | --- | --- | --- | --- | --- | --- |
| PRJNA685612 | Armillaria borealis mycovirgavirus 1 | DN1538_c0_g2_i1^e^ | complete | 38,718 | 344 | 11,213 | Acidomyces richmondensis tobamo-like virus 1 (Gilbert et al., 2019) | 2e-88 | 29 | 38 |
| PRJNA685612 | Armillaria mellea negative strand RNA virus 1 | DN17982_c0_g2_i1^f^ | complete | 35,990 | 336 | 10,795 | Lentinula edodes negative strand RNA virus 1 (Lin et al., 2019) | 0 | 35 | 52 |
| PRJNA685612 | Armillaria mellea ourmia-like virus 1 | DN118_c0_g2_i1 | complete | 19,451 | 435 | 4,426 | Agaricus bisporus virus 15 (Deakin et al., 2017) | 1e-95 | 37 | 41 |
| PRJNA685612 | Armillaria mellea ourmia-like virus 2 | DN8815_c0_g1_i1 | complete | 183,831 | 5719 | 3,199 | Apple ourmia-like virus 3 (GenBank ID QIC52830.1, unpublished) | 6e-74 | 38 | 38 |
| PRJNA685612 | Armillaria ambi-like virus 1 | DN417_c0_g1_i1 | complete | 75,198^d^ | 1410^d^ | 5,235 | Tulasnella ambivirus 4 (Sutela et al., 2020) | 1e-41 | 30 | 39 |
| PRJNA685612 | Armillaria ambi-like virus 2 | DN562_c0_g1_i7 | partial | 282,025^d^ | 5591^d^ | 4,941 | Rhizoctonia solani ambivirus 1 (Forgia et al., 2021) | 1e-100 | 33 | 41 |
| PRJNA685612 | Armillaria ambi-like virus 3 | DN2538_c0_g1_i5 | complete | 188,615^d^ | 4131^d^ | 4,535 | Rhizoctonia solani ambivirus 1 (Forgia et al., 2021) | 3e-101 | 36 | 44 |
| SRR10392772 | Armillaria mellea negative strand RNA virus 2 | DN10343_c0_g1_i1 | partial | 82,197 | 1752 | 8,524 | Bondarzewia berkeleyi negative-strand RNA virus 1 (Vainio and Sutela, 2020) | 0 | 35 | 62 |
| SRR10392772 | Armillaria mellea ambi-like virus 1 | DN6515_c0_g1_i1 | complete | 11,112 | 303 | 5,352 | Cryphonectria parasitica ambivirus 1 (Forgia et al., 2021) | 1e-51 | 29 | 36 |
| SRR2545913 | Armillaria mellea ambi-like virus 2 | DN2557_c0_g1_i2 | complete | 13,912 | 226 | 4,490 | Rhizoctonia solani ambivirus 1 (Forgia et al., 2021) | 1e-104 | 37 | 44 |
| SRR7968262 | Armillaria ectypa ambi-like virus 1 | DN2378_c0_g2_i1 | partial | 2,495 | 151 | 5,035 | Agaricus bisporus (Deakin et al., 2017) | 1e-19 | 26 | 43 |
| SRR7777675 | Armillaria luteobubalina ambi-like virus 1 | DN7471_c0_g1_i3 | partial | 1,876 | 103 | 2,614 | Tulasnella ambivirus 4 (Sutela et al., 2020) | 4e-75 | 31 | 68 |
| SRR13091473 | Armillaria novae-zelandiae ambi-like virus 1 | DN19660_c0_g1_i1 | partial | 361 | 21 | 2,413 | Tulasnella ambivirus 4 (Sutela et al., 2020) | 2e-91 | 32 | 45 |

^a^raw reads were mapped against the contig using Geneious for RNA Seq assembler with medium-low sensitivity

^b^mean read coverage value generated by Geneious 10.2.6

^c^the accession having highest identity in the Blastx search of NCBI nr database

^d^mapping with custom sensitivity using 0 as a maximum mismatch % per read

^e^another almost full-length contig for Armillaria borealis mycovirgavirus 1 was DN1538_c0_g1_i1

^f^another almost full-length contig for Armillaria mellea negative strand RNA virus 1 was DN17982_c0_g1_i1

**Supplementary Table S6.** Variant calling for Armillaria mellea ourmia-like virus 1 (AmOlV1), Armillaria mellea ourmia-like virus 2 (AmOlV2), Armillaria borealis mycovirgavirus 1 (AbMV1) and Armillaria mellea negative strand RNA virus 1 (AmNSRV1)

| ***Armillaria* virus** | **Site** | **Length** | **Change** | **Coverage** | **Polymorphism Type** | **Variant Frequency** | **Variant P-Value (approximate)** | **Average Quality** | **Strand-Bias** | **Variant Raw Frequency** |
| --- | --- | --- | --- | --- | --- | --- | --- | --- | --- | --- |
| **AmOlV1** (ORF region) | 1283 | 1 | G -> A | 177 | SNP (transition) | 29,90 % | 3,30E-135 | 35 | 88,70 % | 53 |
|  | 1531 | 1 | G -> A | 357 | SNP (transition) | 8,40 % | 3,80E-68 | 37 | 60,00 % | 30 |
|  | 1720 | 1 | T -> C | 274 | SNP (transition) | 5,80 % | 4,30E-28 | 34 | 50,00 % | 16 |
|  | 1732 | 1 | C -> T | 351 | SNP (transition) | 16,20 % | 1,40E-139 | 37 | 64,90 % | 57 |
|  | 3071 | 1 | A -> G | 245 | SNP (transition) | 6,50 % | 1,20E-33 | 36 | 62,50 % | 16 |
|  | 3091 | 1 | C -> T | 310 | SNP (transition) | 9,00 % | 7,90E-62 | 36 | 53,60 % | 28 |
|  | 3125 | 1 | C -> T | 361 | SNP (transition) | 6,90 % | 2,10E-47 | 35 | 60,00 % | 25 |
|  |  |  |  |  |  |  |  |  |  |  |
| **AmOlV2** |  |  |  |  |  |  |  |  |  |  |
|  | 1241 | 1 | T -> C | 6707 | SNP (transition) | 15,70 % | 0 | 36 | 50,20 % | 1053 |
|  | 1331 | 1 | T -> A | 5141 | SNP (transversion) | 15,20 % | 0 | 36 | 54,70 % | 782 |
|  | 1762 | 1 | G -> A | 4967 | SNP (transition) | 15,80 % | 0 | 36 | 68,40 % | 786 |
|  | 2023 | 1 | C -> T | 6136 | SNP (transition) | 10,50 % | 0 | 36 | 51,90 % | 645 |
|  | 2143 | 1 | A -> G | 4234 | SNP (transition) | 5,90 % | 0 | 36 | 61,40 % | 251 |
|  |  |  |  |  |  |  |  |  |  |  |
| **AbMV1** |  |  |  |  |  |  |  |  |  |  |
|  | 173 | 1 | G -> A | 148 | SNP (transition) | 5,40 % | 1,20E-17 | 37 | 62,50 % | 8 |
|  | 3199 | 1 | T -> C | 159 | SNP (transition) | 7,50 % | 1,40E-27 | 37 | 66,70 % | 12 |
|  | 4600 | 1 | T -> C | 180 | SNP (transition) | 31,10 % | 1,80E-149 | 36 | 62,50 % | 56 |
|  |  |  |  |  |  |  |  |  |  |  |
| **AmNSV**1 |  |  |  |  |  |  |  |  |  |  |
|  | 4132 | 1 | A -> G | 263 | SNP (transition) | 15,20 % | 3,20E-101 | 37 | 60,00 % | 40 |

Variants were detected using Geneious R10 using minimum variant frequency of 0.05 and maximum P-value of 10e-2. SNPs with 100% strand bias or putative indels at tandem repeat sites were omitted from the list

| **Mycovirus** | **GenBank ID** | **Length (nt)** | **G + C –content %** | **Mapping reads^a^** | **Mean depth^b^** |
| --- | --- | --- | --- | --- | --- |
| Armillaria borealis ambi-like virus 1 strain Ab9A | MW423805 | 4,972 | 48.4 | 23,678 | 466 |
| Armillaria borealis ambi-like virus 2 strain Ab2B | MW423807 | 4,526 | 49.7 | 37,270 | 802 |
| Armillaria borealis ambi-like virus 2 strain Ab4B | MW423808 | 4,526 | 49.0 | 117,692 | 2,533 |
| Armillaria borealis ambi-like virus 2 strain Ab9A | MW423809 | 4,525 | 49.5 | 107,754 | 2,320 |
| Armillaria borealis ambi-like virus 2 strain MUS36 | MW423810 | 4,530 | 49.5 | 130,613 | 2,809 |
| Armillaria sp. ambi-like virus 3 strain Ab9A | MW423812 | 4,523 | 49.8 | 242,567 | 5,225 |
| Armillaria sp. ambi-like virus 3 strain Al65A | MW423813 | 4,526 | 50.6 | 185,123 | 3,985 |

**Supplementary Table S7.** Armillaria borealis ambi-like virus 1, Armillaria borealis ambi-like virus 2 and Armillaria sp. ambi-like virus 3 hosted by *A. borealis* (Ab2B, Ab4B, Ab9A, MUS36) and *A. cepistipes* (Al65A) isolates

^a^raw reads were mapped against the contig using Geneious for RNA Seq assembler with custom sensitivity using 0 as a maximum mismatch % per read

^b^mean read coverage value generated by Geneious 10.2.6

| **Mycovirus** | **Reference^a^** | **Genomic library 1** | | | **Genomic library 2** | | | **Genomic library 3** | | |
| --- | --- | --- | --- | --- | --- | --- | --- | --- | --- | --- |
|  |  | **BioProject** | **SRA** | **Number of significant hits** | **BioProject** | **SRA** | **Number of significant hits** | **BioProject ID** | **SRA ID** | **Number of significant hits** |
| Armillaria mellea negative strand RNA virus 2 | JGI, Francis M. Martin | PRJNA571622 | SRR10392760 | 0 | PRJNA571622 | SRR10392759 | 0 | PRJNA571622 | SRR10392758 | 0 |
| Armillaria ectypa ambi-like virus 1 | JGI, Francis M. Martin | PRJNA463936 | SRR7968115 | 0 | PRJNA463936 | SRR7968114 | 0 | NA | NA | NA |
| Armillaria luteobubalina ambi-like virus 1 | JGI, Jonathan M. Plett | PRJNA500536 | SRR8239694 | 0 | PRJNA500536 | SRR8239693 | 0 | PRJNA500536 | SRR8239692 | 0 |
| Armillaria mellea ambi-like virus 1 | JGI, Francis M. Martin | PRJNA571622 | SRR10392760 | 0 | PRJNA571622 | SRR10392759 | 0 | PRJNA571622 | SRR10392758 | 0 |
| Armillaria mellea ambi-like virus 2 | NA | NA | NA | NA | NA | NA | NA | NA | NA | NA |
| Armillaria novae-zelandiae ambi-like virus 1 | JGI, Francis M. Martin | PRJNA677794 | SRR13091472 | 0 | PRJNA677794 | SRR13091471 | 0 | NA | NA | NA |

**Supplementary Table S8.** The BioProject and SRA run IDs (NCBI) utilized in Blastn analyzes conducted in order to determine the possibility of virus integration to host genome

^a^Contact person of unpublished data of JGI (Joint Genome Institute)

**Supplementary Table S9.** Primers utilized in the current study

| **Target amplicon** | **Purpose** | **Trinity contig** | **Forward primer** | **Sequence of forward primer** | **Reverse primer** | **Sequence of reverse primer** |
| --- | --- | --- | --- | --- | --- | --- |
| Armillaria borealis mycovirgavirus 1 | Screening of host isolates | DN1538_c0_g2_i1 (reversed) | Armllr-Tobamo-DN1538-F | GCTACGGAAAAACCGATGAA | Armllr-Tobamo-DN1538-R | CTGAAACATCTCGCGCATTA |
| Armillaria mellea mymona-like | Screening of host isolates | DN17982_c0_g2_i1 | Armllr-Mymona-DN17982-F | GAACTGAAGCCGGAAGAGTG | Armllr-Mymona-DN17982-R | GCTAGGGGAGACTGGCTTCT |
| Armillaria ourmia 1 | Screening of host isolates | DN118_c0_g2_i1 (reversed) | Armllr-Ourmia1-DN118-F | GACCCACGTCTGAAAGGAAA | Armllr-Ourmia1-DN118-R | CATGTCCAACCAACTCAACG |
| Armillaria ourmia 2 | Screening of host isolates | DN8815_c0_g1_i1 (reversed) | Armllr-Ourmia2-DN8815-F | GCTTGTCTGCTAGGGTCAGG | Armllr-Ourmia2-DN8815-R | AATTCCCCATCATTTGACCA |
| Armillaria ambi-like 1 | Screening of host isolates | DN417_c0_g1_i1 | Armllr-AS3-DN417-F | TGTGCCACGATGAACGTATT | Armllr-AS3-DN417-R | GGGGATGTCCTTTCCAAAAT |
| Armillaria ambi-like 2 | Screening of host isolates | DN741_c0_g1_i9 | Armllr-AS2-screenF | TTCGGAGATACGCAGATCG | Armllr-AS2-screenR | CGAAATGGTGGACTTCTTCC |
| Armillaria ambi-like 3 | Screening of host isolates | DN2538_c0_g1_i5 (reversed) | Armllr-AS3-DN2538-F | AGGTGTGGGTTTGACTCTGG | Armllr-AS3-DN2538-R | ATCCCGCATATGGTAGCAAG |
| ORFan DN11424 | Screening of host isolates | DN11424_c0_g1_i1 | Armllr-DN11424-F | AGAAATCGATCCGCTCTTCA | Armllr-DN11424-R | CCACTGTCCAACTTCCCCTA |
| Armillaria borealis mycovirgavirus 1 | Determination of 5' UTR region | DN1538_c0_g2_i1 (reversed) | T4 RNA primer^a^ or primer A^b^ |  | Armllr-Tobamo-RTR | ATATTACGTTTGTTGCGCCT |
| Armillaria borealis mycovirgavirus 1 | Determination of 3' UTR region | DN1538_c0_g2_i1 (reversed) | Armllr-Tobamo-RTF | ACTATGGAGGCGTTGATGGC | T4 RNA primer^a^ or primer A^b^ |  |
| Armillaria mellea mymona-like | Determination of 5' UTR region | DN17982_c0_g2_i1 | T4 RNA primer^a^ or primer A^b^ |  | Armllr-Mymona-RTR | GGCTCCTCTGGACGAACATC |
| Armillaria mellea mymona-like | Determination of 3' UTR region | DN17982_c0_g2_i1 | Armllr-Mymona-RTF | GAACTGATACAAGACCTCGC | T4 RNA primer^a^ or primer A^b^ |  |
| Armillaria ourmia 1 | Determination of 5' UTR region | DN118_c0_g2_i1 (reversed) | T4 RNA primer^a^ or primer A^b^ |  | Armllr-Ourmia1-RTR | CGCTCTCCATTTCGTCACTG |
| Armillaria ourmia 1 | Determination of 5' UTR region | DN118_c0_g2_i1 (reversed) | T4 RNA primer^a^ or primer A^b^ |  | Armllr-Ourmia1-RTR2 | TCCAGGAACACACAGTCTGC |
| Armillaria ourmia 1 | Determination of 5' UTR region | DN118_c0_g2_i1 (reversed) | T4 RNA primer^a^ or primer A^b^ |  | Armllr-Ourmia1-RTR3 | TGTCGTGGTTTCTTCTCGTG |
| Armillaria ourmia 1 | Determination of 3' UTR region | DN118_c0_g2_i1 (reversed) | Armllr-Ourmia1-RTF | CCTATTGTGGAAGACGTCGC | T4 RNA primer^a^ or primer A^b^ |  |
| Armillaria ourmia 1 | Determination of 3' UTR region | DN118_c0_g2_i1 (reversed) | Armllr-Ourmia1-RTF2 | CAGGAAGTTGTCACGGGATT | T4 RNA primer^a^ or primer A^b^ |  |
| Armillaria ourmia 1 | Validation of contig 5' region | DN118_c0_g2_i1 (reversed) | Armllr-Ourmia1-BegF | GTGAGAAGCCTCGAAGGATG |  |  |
| Armillaria ourmia 2 | Determination of 5' UTR region | DN8815_c0_g1_i1 (reversed) | T4 RNA primer^a^ or primer A^b^ |  | Armllr-Ourmia2-RTR | CTTGACAACGCTGAACACCA |
| Armillaria ourmia 2 | Determination of 5' UTR region | DN8815_c0_g1_i1 (reversed) | T4 RNA primer^a^ or primer A^b^ |  | Armllr-Ourmia2-RTR2 | AGTTTTCCACGCTCTTTCCA |
| Armillaria ourmia 2 | Determination of 3' UTR region | DN8815_c0_g1_i1 (reversed) | Armllr-Ourmia2-RTF | TTAACTCTTTCACGCCACGC | T4 RNA primer^a^ or primer A^b^ |  |
| Armillaria ourmia 2 | Determination of 3' UTR region | DN8815_c0_g1_i1 (reversed) | Armllr-Ourmia2-RTF2 | CGCGAAGTGGCTTTTTACTC | T4 RNA primer^a^ or primer A^b^ |  |
| Armillaria ourmia 2 | Validation of contig 5' region | DN8815_c0_g1_i1 (reversed) | Armllr-Ourmia2-BegF | GGCCTAACCGGTTCAACTCTT |  |  |
| Armillaria ambi-like 1 | Amplification of virus genome | DN417_c0_g1_i1 | Armllr-AS1-MidF | TTAAGCCTCCACCCCCTACT | Armllr-AS1-EndR | TTGGCACTCGAGATTCTTCA |
| Armillaria ambi-like 1 | Amplification of virus genome | DN417_c0_g1_i2 | Armllr-AS1-BegF | GAAATCCGTGTTCCTGGTTG | Armllr-AS1-MidR | TTGGTTGGTGATGGTGTGAT |
| Armillaria ambi-like 1 | Amplification of virus genome | DN417_c0_g1_i3 | Armllr-AS1-RTF2 | CTTTGGGAACAGGACCTTCT | Armllr-AS1-RTR2 | CGGGAAGGCCTTTTTCTTT |
| Armillaria ambi-like 1 | Determination of 5' UTR region | DN417_c0_g1_i4 | T4 RNA primer^a^ or primer A^b^ |  | Armllr-AS1-RTR2 | CGGGAAGGCCTTTTTCTTT |
| Armillaria ambi-like 1 | Determination of 3' UTR region | DN417_c0_g1_i5 | Armllr-AS1-RTF2 | CTTTGGGAACAGGACCTTCT | T4 RNA primer^a^ or primer A^b^ |  |
| Armillaria ambi-like 1 | Amplification of virus genome | DN417_c0_g1_i6 | Armllr-AS1-Ab9aF | TTAAGCCTCCACCCCCTACT | Armllr-AS1-Ab9aR | GGATCTTTGGCACTCGAGAC |
| Armillaria ambi-like 1 | Primer utilized in Sanger sequencing | DN417_c0_g1_i7 |  |  | Armllr_AS1_BegR | GAAGACCAAATTGTGCTCATAGG |
| Armillaria ambi-like 1 | Primer utilized in Sanger sequencing | DN417_c0_g1_i8 |  |  | Armllr-AS1-Rev | TGTCGTCTTTTATGCGCAAG |
| Armillaria ambi-like 2 | Amplification of virus genome | DN741_c0_g1_i9 | Armllr-AS2-BegF | ATCCGAGTGTCTTGCTCAGG | Armllr-AS2-MidR | CTTGAGCACCCTCAATGGTT |
| Armillaria ambi-like 2 | Amplification of virus genome | DN741_c0_g1_i10 | Armllr-AS2-MidF | CTTGGCAAGCTTTGATCAGG | Armllr-AS2-EndR | AATTCTCGGTTGACCACCAC |
| Armillaria ambi-like 2 | Amplification of virus genome | DN741_c0_g1_i11 | Armllr-AS2-RTF2 | GCCAGTTCGATAATCCATGC | Armllr-AS2-RTR2 | TTCTTGGAGACTCCCCCATT |
| Armillaria ambi-like 2 | Determination of 5' UTR region | DN741_c0_g1_i12 | T4 RNA primer^a^ or primer A^b^ |  | Armllr-AS2-RTR2 | TTCTTGGAGACTCCCCCATT |
| Armillaria ambi-like 2 | Determination of 3' UTR region | DN741_c0_g1_i13 | Armllr-AS2-RTF2 | GCCAGTTCGATAATCCATGC | T4 RNA primer^a^ or primer A^b^ |  |
| Armillaria ambi-like 2 | Primer utilized in Sanger sequencing | DN741_c0_g1_i14 | Armllr_AS2_BegF2 | GGAAGGTCCTCCCATACG |  |  |
| Armillaria ambi-like 2 | Primer utilized in Sanger sequencing | DN741_c0_g1_i15 |  |  | Armllr_AS2_BegR | CGAGATGAGGCAGAAGTGGT |
| Armillaria ambi-like 2 | Primer utilized in Sanger sequencing | DN741_c0_g1_i16 | Armllr_AS2_EndF | TTCATGCTTGCTCGTGAGAC |  |  |
| Armillaria ambi-like 3 | Amplification of virus genome | DN2538_c0_g1_i5 (reversed) | Armllr-AS3-BegF | GCACGAGTGGCTGGTTACTT | Armllr-AS3-MidR | TTCTCGAAATACCGGAGCAC |
| Armillaria ambi-like 3 | Amplification of virus genome | DN2538_c0_g1_i5 (reversed) | Armllr-AS3-MidF | CGGGGTACACCAGAGAGAAG | Armllr-AS3-EndR | TCGGCACAATCACAATTCAG |
| Armillaria ambi-like 3 | Amplification of virus genome | DN2538_c0_g1_i5 (reversed) | Armllr-AS3-DN2538-RTF | CCTGGAGGCAAGTTGATGAT | Armllr-AS3-DN2538-RTR | TACGTGATGTACCCGAAGCA |
| Armillaria ambi-like 3 | Determination of 5' UTR region | DN2538_c0_g1_i5 (reversed) | T4 RNA primer^a^ or primer A^b^ |  | Armllr-AS3-DN2538-RTR | TACGTGATGTACCCGAAGCA |
| Armillaria ambi-like 3 | Determination of 3' UTR region | DN2538_c0_g1_i5 (reversed) | Armllr-AS3-DN2538-RTF | CCTGGAGGCAAGTTGATGAT | T4 RNA primer^a^ or primer A^b^ |  |
| Armillaria ambi-like 3 | Primer utilized in Sanger sequencing | DN2538_c0_g1_i5 (reversed) | Armllr-AS3-BegF2 | GCGCATGATCCTACTAGGTC |  |  |
| Armillaria ambi-like 3 | Primer utilized in Sanger sequencing | DN2538_c0_g1_i5 (reversed) |  |  | Armllr_AS3_EndR2 | CCGGATTATGGTCTTTGACG |
| Armillaria ambi-like 3 | Primer utilized in Sanger sequencing | DN2538_c0_g1_i5 (reversed) | Armllr-AS3-EndF | CATAGTGGGGGAAGGCTACA |  |  |
| Armillaria ambi-like 3 | Primer utilized in Sanger sequencing | DN2538_c0_g1_i5 (reversed) |  |  | Armllr-AS3-Rev | ACCGTACACARGGCTCAATC |

^a^Modified after Lambden et al. (1992) by Tuomivirta and Hantula (2003).

^b^Adapter sequence as described in Attoui et al. (2000)


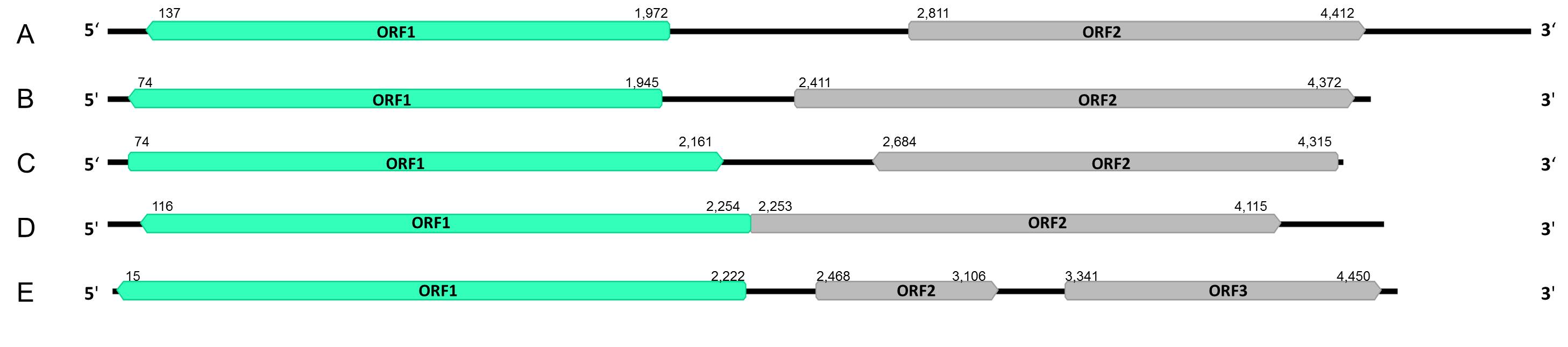


**Supplementary Fig. S1.** Genome organization of the ambi-like viruses retrieved from *Armillaria* RNAseq libraries *in silico*. A) Armillaria ectypa ambi-like virus 1 strain FPL83.16 (TPA: BK014418). B) Armillaria luteobubalina ambi-like virus 1 strain HWK02 (TPA: BK014419). C) Armillaria mellea ambi-like virus 1 strain ELDO17 (TPA: BK014420). D) Armillaria mellea ambi-like virus 2 (TPA: BK014421). E) Armillaria novae-zelandiae ambi-like virus 1 strain 2840 (TPA: BK014422). Predicted translation initiation and termination sites of ORFs are marked above or below each ORF. ORF1(coloured with green hue) is a putative RNA-dependent RNA polymerase (contains GDD motif).


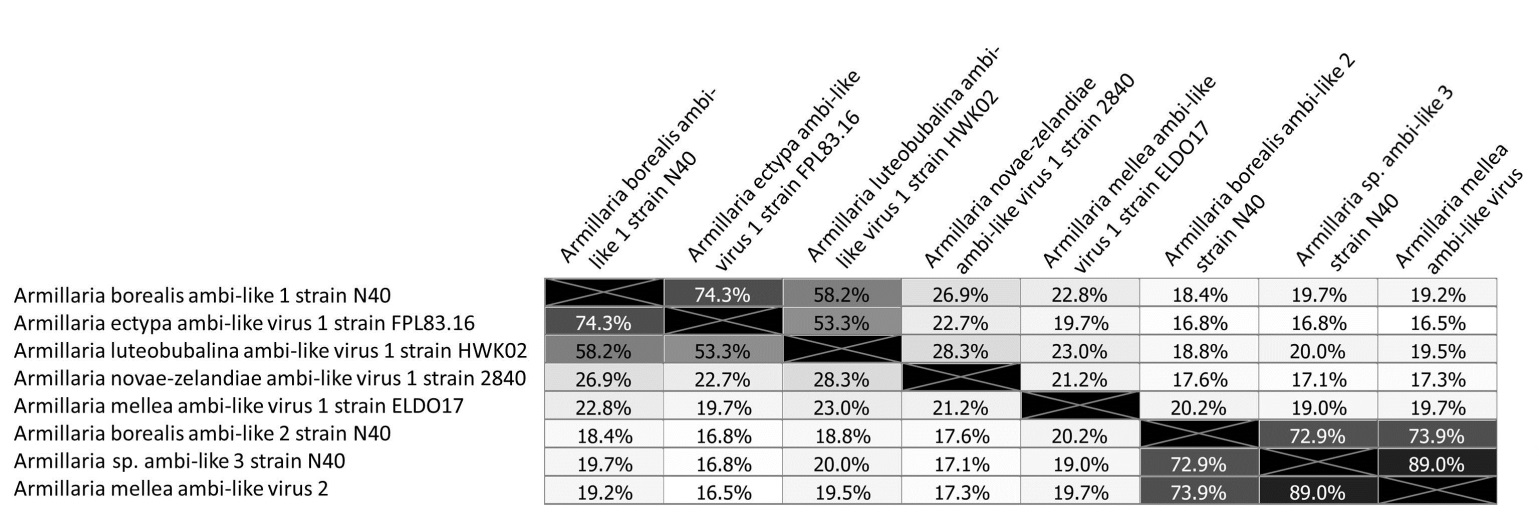


**Supplementary Fig. S2.** Pairwise identities of ambi-like viruses hosted by *Armillaria* sp. Each cell presents pairwise identities between the predicted aa of putative RdRPs of ambi-like viruses Armillaria borealis ambi-like 1 strain N40 (MW423804), Armillaria ectypa ambi-like virus 1 strain FPL83.16 (TPA: BK014418), Armillaria luteobubalina ambi-like virus 1 strain HWK02 (TPA: BK014419), Armillaria novae-zelandiae ambi-like virus 1 strain 2840 (TPA: BK014422), Armillaria mellea ambi-like virus 1 strain ELDO17 (TPA: BK014420), Armillaria borealis ambi-like 2 strain N40 (MW423806), Armillaria sp. ambi-like 3 strain N40 (MW423811) and Armillaria mellea ambi-like virus 2 (TPA: BK014421). Alignments were generated with MAFFT in Geneious 10.2.6.


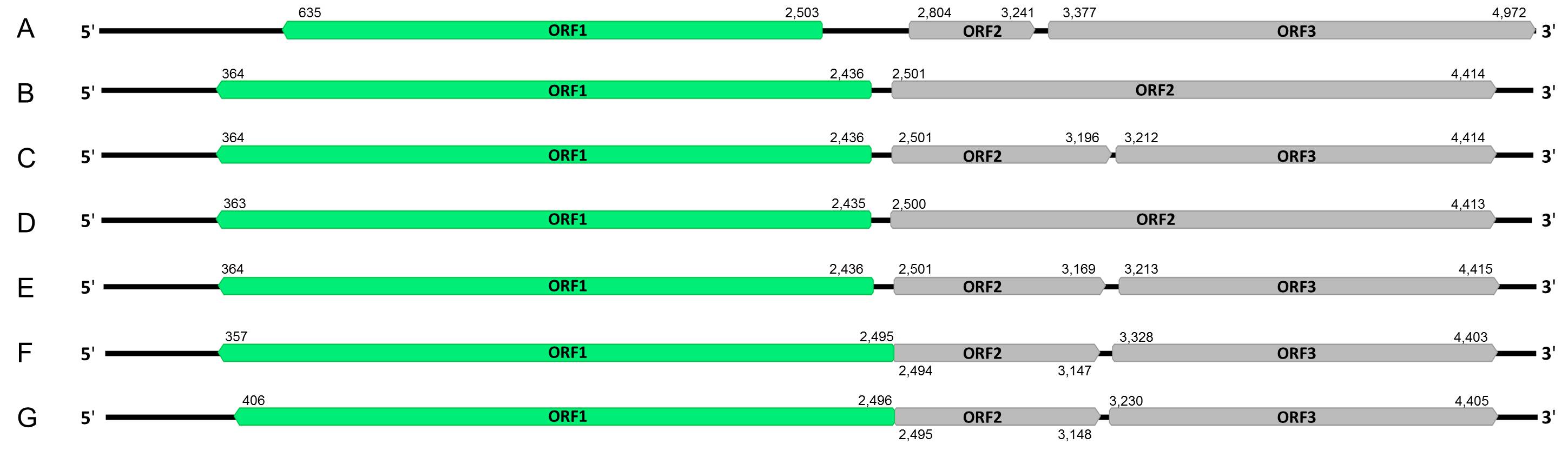


**Supplementary Fig. S3.** Genome organization of the ambi-like viruses hosted by *Armillaria* *borealis* and *A*. *cepistipes*. A) Armillaria borealis ambi-like virus 1 strain Ab9A (MW423805). B) Armillaria borealis ambi-like virus 2 strain Ab2B (MW423807). C) Armillaria borealis ambi-like virus 2 strain Ab4B (MW423808). D) Armillaria borealis ambi-like virus 2 strain Ab9A (MW423809). E) Armillaria borealis ambi-like virus 2 strain MUS36 (MW423810). F) Armillaria sp. ambi-like virus 3 strain Ab9A (MW423812). G) Armillaria sp. ambi-like virus 3 strain Al65A (MW423813). Predicted translation initiation and termination sites of ORFs are marked above or below each ORF. ORF1(coloured with green hue) is a putative RNA-dependent RNA polymerase (contains GDD motif).

**References of Supplementary data**

Attoui, H., Billoir, F., Cantaloube, J. F., Biagini, P., de Micco, P. & de Lamballerie, X. Strategies for the sequence determination of viral dsRNA genomes. *J. Virol. Methods.* **89**, 147-158 (2000).

Deakin, G., Dobbs, E., Bennett, J. M., Jones, I. M., Grogan, H. M. & Burton, K. S. Multiple viral infections in *Agaricus bisporus* – Characterisation of 18 unique RNA viruses and 8 ORFans identified by deep sequencing. *Sci. Rep.* **7**, 2469; 10.1038/s41598-017-01592-9 (2017).

Forgia, M., Isgandarli, E., Aghayeva, D. N., Huseynova, I. & Turina, M. Virome characterization of *Cryphonectria parasitica* isolates from Azerbaijan unveiled a new mymonavirus and a putative new RNA virus unrelated to described viral sequences. *Virology* **553**, 51-61 (2021).

Fradj, N. et al. A first insight into North American plant pathogenic fungi *Armillaria sinapina* transcriptome. Biology **9**, 153; 10.3390/biology9070153 (2020).

Gilbert, K. B., Holcomb, E. E., Allscheid, R. L. & Carrington, J. C. Hiding in plain sight: New virus genomes discovered via a systematic analysis of fungal public transcriptomes. *PLoS One* **14**, e0219207; 10.1371/journal.pone.0219207 (2019).

Lambden, P. R., Cooke, S. J., Caul, E. O. & Clarke, I. N. Cloning of noncultivatable human rotavirus by single primer amplification. *J. Virol*. **66**, 1817-1822 (1992).

Lei, M. et al. Complete genome sequence of *Paenibacillus polymyxa* CF05, a strain of plant growth-promoting Rhizobacterium with elicitation of induced systemic resistance. *Genome Announc*. **3** :e00198-15; 10.1128/genomeA.00198-15 (2015).

Lin, Y. H. et al. Two novel fungal negative-strand RNA viruses related to mymonaviruses and phenuiviruses in the shiitake mushroom (*Lentinula edodes*). *Virology* **533**, 125-136 (2019).

Sahu, N. et al. Hallmarks of basidiomycete soft- and white-rot in wood-decay -omics data of *Armillaria*. bioRxiv 2020.05.04.075879; /10.1101/2020.05.04.075879 (2020).

Sutela, S. et al. The virome from a collection of endomycorrhizal fungi reveals new viral taxa with unprecedented genome organization. *Virus Evol.* **6**, veaa076; 10.1093/ve/veaa076 (2020).

Vainio, E. J. & Sutela, S. Mixed infection by a partitivirus and a negative-sense RNA virus related to mymonaviruses in the polypore fungus *Bondarzewia berkeleyi*. Virus Res. **286**, 198079; 10.1016/j.virusres.2020.198079 (2020).

Tsai, C. C. et al. Comparative transcriptome analysis of *Gastrodia elata* (Orchidaceae) in response to fungus symbiosis to identify gastrodin biosynthesis-related genes. *BMC Genomics* **17**, 212; 10.1186/s12864-016-2508-6 (2016).

Tuomivirta, T. T. & Hantula, J. *Gremmeniella abietina* mitochondrial RNA virus S1 is phylogenetically related to the members of the genus Mitovirus. *Arch. Virol.* **148**, 2429-2436 (2003).

Zhan, M. et al. Draft genomic sequence of *Armillaria gallica* 012m: insights into its symbiotic relationship with *Gastrodia elata*. *Braz. J. Microbiol*. **51**, 1539-1552 (2020).
